# A novel ETV6-miR-429-CRKL regulatory circuitry contributes to aggressiveness of hepatocellular carcinoma

**DOI:** 10.1186/s13046-020-01559-1

**Published:** 2020-04-23

**Authors:** Chunmei Guo, Chao Gao, Dongting Zhao, Jiahui Li, Jinxia Wang, Xujuan Sun, Qinlong Liu, Lihong Hao, Frederick T. Greenaway, Yuxiang Tian, Shuqing Liu, Ming-Zhong Sun

**Affiliations:** 1 Department of Biotechnology, College of Basic Medical Sciences, Dalian Medical University, Dalian, 116044 China; 2 Department of Biochemistry, College of Basic Medical Sciences, Dalian Medical University, Dalian, 116044 China; 3 Department of General Surgery, The Second Affiliated Hospital, Dalian Medical University, Dalian, 116044 China; 4 Department of Histology and Embryology, College of Basic Medical Sciences, Dalian Medical University, Dalian, 116044 China; 5 Carlson School of Chemistry and Biochemistry, Clark University, Worcester, MA 01610 USA

**Keywords:** Hepatocellular carcinoma, ETV6-miR-429-CRKL regulatory circuitry, Malignancy

## Abstract

**Background:**

Tumor metastasis is one of the main causes of the high mortality of hepatocellular carcinoma (HCC). E-Twenty Six variant gene 6 (ETV6) is a strong transcriptional repressor, associated with the development and progression of tumors. However, the exact role and underlying mechanism of ETV6 in HCC remain unclear.

**Methods:**

Western blotting, quantitative real-time PCR and immunohistochemistry *were used to* detect the expression levels of ETV6, CRKL (v-crk sarcoma virus CT10 oncogene homologue (avian)-like) and miR-429 in HCC tissues and cells; Transwell chamber and F-actin cytoskeleton staining assay to examine the effects of ETV6 and CRKL deregulation on the migration, invasion and cytoskeleton of HCC cells; Co-immunoprecipitation assay to determine the interaction between CRKL and ETV6; Chromatin immunoprecipitation assay to investigate the interaction between ETV6 and miR-429.

**Results:**

We established a novel ETV6-miR-429-CRKL regulatory circuitry contributes to HCC metastasis. ETV6 and CRKL were frequently increased, while miR-429 was downregulated in both hepatocarcinoma tissues and hepatocarcinoma cells. Moreover, ETV6 upregulation was positively correlated with CRKL upregulation, and two negative correlations were also established for ETV6 and CRKL upregulation with miR-429 downregulation in both hepatocarcinoma patients’ tumorous tissues and hepatocarcinoma cells. Functional investigations revealed that overexpression and knockdown of ETV6 was remarkably effective in promoting and suppressing HCC cell migration, invasion, cytoskeleton F-actin expression and arrangement, whereas, CRKL overexpression exhibited similar effects to the overexpression of ETV6. Mechanistically, ETV6 negatively regulates miR-429 expression by directly binding to the promoter region of miR-429; miR-429 negatively regulates CRKL expression by selectively targeting *CRKL*-3′-UTR; ETV6 directly binds to CRKL and positively regulates its expression, which in turn CRKL positively regulates ETV6 expression.

**Conclusions:**

Our data demonstrated that ETV6 promotes migration and invasion of HCC cells by directly binding to promoter region of miR-429 via modulating CRKL expression. The newly identified ETV6-miR-429-CRKL regulatory circuitry contributes to the aggressiveness of HCC, which provides new clues for fundamental research on diagnosis and treatment parameters for HCC.

## Background

Hepatocellular carcinoma (HCC) is one of the most common cancers worldwide. Its morbidity and mortality rate are among the highest in the world [1, 2]. Tumor metastasis is the major problem leading to its high recurrence with low post-surgical 5-years survival and high mortality [3–5]. It is a multistep process, including the invasion of extracellular matrix (ECM), intravasation, translocation, migration and invasion of a secondary site, and finally the formation of metastatic nodules [3–5]. Deeper study of the molecular mechanisms of HCC metastasis can lead to novel therapeutic targets and improve the prognosis for HCC patients.

E-Twenty Six (ETS) variant gene 6 (ETV6), also known as translocation ets leukaemia (Tel), is a member of the transcription factor ETS family [6]. It is composed of a helix-loop-helix (HLH) domain [also named as pointed domain (PNT), or sterile alpha motif domain (SAM)] at the N-terminus and an ETS domain at the C-terminus. The HLH domain is necessary for homodimerization and heterodimerization with other ETS family members, it also frequently contributes to fusion proteins as the partner of tyrosine kinases [7, 8]. The ETS domain is responsible for sequence specific DNA-binding to the ETS-binding consensus site (EBS) [6–8]. ETV6 is known to form fusion proteins by chromosomal translocations with the platelet-derived growth factor receptor (PDGFR), v-abl Abelson murine leukemia viral oncogene (ABL), runt-related transcription factor 1 gene (RUXN1), ecotropic virus integration site-1 (EVI-1) through the HLH domain, leading to the homodimerization of fusion proteins and to constitutive activation of tyrosine kinase activity [9]. The HLH domain of ETV6 could mediate dimerization of ABL and subsequent ABL kinase domain activation [10]. CRKL (v-crk sarcoma virus CT10 oncogene homologue (avian)-like) is a substrate protein for ABL, Tel-ABL fusion protein can form complexes with CRKL leading to tyrosine-phosphorylation of CRKL and the constitutive activation of Ras (rat sarcoma), MAPK/ERK (mitogen-activated protein kinase/extracellular regulated protein kinases) and PKB/AKT (protein kinase B/Serine/threonine kinase) [10]. ETV6 also acts as a strong transcriptional repressor by directly binding to target genes through the ETS DNA-binding domain to repress its transcription [11, 12]. ETV6 plays a crucial role in embryonic development and hematopoiesis [13]. Its deregulation is also involved in the development and progression of leukemia, prostate cancer, colorectal cancer and non-small cell lung cancer [14–18]. However, the biological function and underlying regulation mechanism of ETV6 in HCC remain unclear.

CRKL, a member of the CRK adapter protein family, is ubiquitously expressed and conserved across eukaryotic organisms [19]. It is composed of one NH2-terminal Src homology2 (SH2) domain, one N-terminal SH3 (SH3N) domain and one C-terminal SH3 (SH3C) domain. CRKL has a variety of linkages for coupling to proline-rich proteins BCAR1 (breast cancer anti-estrogen resistance 1), GAB (Grb2-associated binder protein), ABL, Pax (paired boxed gene), GEF (guanine nucleotide exchange factor), C3G (3-domain-binding guanine nucleotide exchange factor), BCR (B-cell receptor)-ABL and SOS (son of sevenless) to form timely and localized complexes that are critical for cell proliferation, survival, adhesion and migration [20, 21]. Hence it can function in cellular signaling cascades by either directly forms complex with downstream receptor protein to regulate cellular tyrosine kinase activity, or by acting as an upstream mediator for signal initiation [22, 23]. CRKL deregulation has been linked to the development and progression of a variety of cancers [24, 25]. We previously reported that the overexpression and knockdown of CRKL was remarkably effective in promoting and suppressing the in vitro migration and invasion capacities of hepatocarcinoma HepG2 cells [26].

MicroRNAs (miRNAs) are 18–24 nucleotide small non-coding RNAs that negatively regulate gene expression by directly degrading mRNA or by suppressing post-transcriptional protein translation by binding to the 3′-untranslated region (3′-UTR) of targeted mRNAs [27]. MiRNAs play important roles in cell proliferation, differentiation, metastasis and apoptosis by directly degrading mRNA or by suppressing post-transcriptional protein translation by targeting mRNAs [28, 29]. MiRNAs might function as tumor promoter or suppressor in tumorigenesis and tumor malignancy [30]. miR-429, a member of miR-200 family, is located on chromosome 1p36 [31]. miR-429 dysregulation is involved in the development, invasion, epithelial-mesenchymal transition (EMT), metastasis and drug resistance of various cancers [32, 33]. It functions either as a tumor suppressor or tumor promoter for certain cancers depending on the particular type of tumor cell/tissue [34–36]. Our previous study showed that miR-429 negatively regulated CRKL expression by selectively binding to *CRKL*-3′-UTR at the 3728–3735 bp site by post-transcriptionally mediating CRKL’s functionality, and miR-429 suppressed the migration and invasion of HepG2 cells by targeting CRKL via inhibiting Raf/MEK/ERK (rapidly accelerated fibrosarcoma/ mitogen-activated extracellular signal-regulated kinase/ERK) pathway and EMT [26]. Bioinformatics analysis software JASPAR predicted that ETV6 protein potentially binds to the DNA promoter region at the 696–705 site of miR-429. We speculate that ETV6, CRKL and miR-429 may form an ETV6-miR-429-CRKL regulatory loop.

In the present study, we report a new ETV6-miR-429-CRKL regulatory circuitry in HCC. We found that ETV6 and CRKL were upregulated, and miR-429 was downregulated in HCC patients’ tissues and HCC cell lines compared with corresponding non-tumor liver tissues and a normal liver cell line. Moreover, ETV6 knockdown inhibited the in vitro migration, invasion and F-actin cytoskeleton expression and arrangement of HCC cells, while ETV6 or CRKL overexpression showed opposite effects. We observed that overexpression of ETV6 caused repression of miR-429 and the inhibition of miR-429 lead to the upregulation of CRKL, which in turn CRKL overexpression promoted ETV6 expression. Meanwhile, the expression of ETV6 displayed a positive correlation with CRKL but an inverse correlation with miR-429. Furthermore, ETV6 directly binds to the promoter region at the 696–705 bp site of miR-429 by transcriptionally negatively mediating its expression; CRKL is a direct target of miR-429 and miR-429 negatively regulates CRKL expression by selectively binding to *CRKL*-3′-UTR at the 3728–3735 bp site by post-transcriptionally mediating its functionality; ETV6 positively regulates CRKL expression by directly cooperating with CRKL. Our study uncovered a novel ETV6-miR-429-CRKL regulatory circuitry in maintaining the aggressive phenotypes of HCC and partially elucidated the molecular mechanism of HCC metastasis.

## Materials and methods

### Patients and tissue samples

Sixteen pairs of matched tumor tissues and corresponding non-tumor liver tissues from hepatocarcinoma patients were collected from the Division of Hepatobiliary and Pancreatic Surgery, Department of Surgery, The Second Affiliated Hospital of Dalian Medical University, Dalian, China. None of the patients had received radiotherapy or chemotherapy before tissue collection. There were 11 men and 5 women, 7 patients of age ≥ 60 years and 9 patients of age < 60 years; 8, 4 and 4 patients are cataloged into T1, T2 and T3 stages; 12 patients are primary HCC and 4 patients unknown; 1 patient exhibits perineuronal invasion, 10 patients no perineuronal invasion and 5 patients unknown; 5 patients have satellite nodules, 4 patients without satellite nodules and 7 patients unknown; 8 patients are HBV (hepatitis B virus) positive and 8 patients unknown; 1 patient of AFP (alpha fetoprotein) ≤ 20 IU/ml, 5 patients of 20 < AFP ≤ 400 IU/ml, 3 patients of AFP>400 IU/ml and 7 patients unknown. Tissue specimens were frozen in liquid nitrogen immediately after surgical resection and stored at − 80 °C until use. The use of human tissues and the study protocol was approved by the Medical Ethics Committee of Dalian Medical University, and informed consent was obtained from all patients. All experiment methods were performed in accordance with the relevant guidelines and regulations.

### Cell culture

Human normal liver LO2, hepatocellular carcinoma HepG2, HuH7, HCCLM3 and human embryonic kidney 293 T cells were purchased from the Shanghai Culture Collection of Chinese Academy of Sciences and cultured in 90% Dulbecco′s modified Eagle′s medium (DMEM, Gibco, USA) supplemented with 10% fetal bovine serum (FBS, TransGen, China), 100 U/ml penicillin and 100 U/ml streptomycin (Gibco, USA) in a humidified incubator at 37 °C with 5% CO_2_.

### Western blotting (WB) assay

Total protein was extracted from each group cells using radio-immunoprecipitation assay (RIPA) buffer [50 mM pH 8.0 Tris-HCl, 150 mM NaCl, 1% Triton X-100, 0.5% sodium deoxycholate, 0.1% SDS in the presence of 1 mM Na_3_VO_4_, 1 μg/ml leupeptin and 0.5 mM PMSF (phenylmethanesulfonyl fluoride)]. The supernatant was collected by centrifugation at 12000 rpm at 4 °C for 15 min. Equal amounts of each group of protein samples determined by Bradford assay were boiled for 5 min in loading buffer and separated by 10% SDS-PAGE (sodium dodecyl sulfate-polyacrylamide gel electrophoresis). The protein bands were transferred onto a nitrocellulose (NC) membrane (PALL, USA), blocked with 5% (w/v) skim milk (BD, USA) in TBST (Tris-buffered-saline-tween 20, pH 7.5, 100 mM NaCl, 50 mM Tris and 0.1% Tween-20) for 3 h at room temperature (RT) and then incubated with primary antibodies at 4 °C overnight. The primary antibodies were CRKL (1:2000, Santa Cruz Biotechnology, USA), ETV6 (1:400, Abcam, USA), ACTB (β-actin, 1:4000, Sanying, China) and GAPDH (glyceraldehyde-3-phosphate dehydrogenase, 1:4000, Proteintech, USA). The NC membrane was then washed with TBST for 3 × 10 min, incubated with the secondary antibody conjugate for 3 h at RT and washed again with TBST 3 × 10 min. Protein bands were visualized by electrochemiluminescence (ECL, Advansta, USA) and analyzed using the Bio-Rad ChemiDoc™ MP system (Bio-Rad, USA). GAPDH and ACTB were used as internal references.

### Immunohistochemistry (IHC) assay

IHC assay was used to determine the expression levels of CRKL (1:2000, Santa Cruz Biotechnology, USA) in HCC tissue arrays. Tissue sections were treated with biotin-streptavidin horseradish peroxidase (HRP) detection systems (ZSGB-BIO, China) according to the manufacturer’s protocol. The images were visualized with a DAB (diaminobenzidin) kit (ZSGB-BIO, China) using a BX63 microscope (Olympus, Japan). IHC immunoreaction intensity was rated into four grades, 0 (negative), 1 (weak), 2 (moderate) and 3 (strong) as Score I. Moreover, based on the detected positively staining cells, DAB staining quantity of each sample was graded as 0 (none), 1 (1–10% cells per field), 2 (10–50%), 3 (51–75%) and 4 (> 76%) as Score II. The multiplication of Score I by Score II ranging from 0 to 12 was used as a measure of IHC immunoreactivity degree. The scores of 0–2, 3–5, 6–8, and 9–12 were considered as negative (−), weak (+), moderate (++) and strong (+++). IHC assays were scored separately by two independent experienced pathologists.

### Quantitative real-time PCR (qRT-PCR) assay

Total RNA was extracted from each group of tissues and cells using Trizol™ reagent (Invitrogen, USA) and reversely transcribed into an EasyScript One-Step gDNA Removal and cDNA Synthesis SuperMix kit (TransGen, China). qRT-PCR was then performed using TransStart Tip Green qPCR SuperMix (TransGen, China) with a StepOnePlus™ Real-Time PCR system (ThermoFisher, USA). snRNA U6 was used as internal reference for miR-429. The relative expression levels of miR-429 in different groups of cells, and in paired tumor and paracancerous non-tumor tissues from hepatocarcinoma patients were compared using the 2^-△△CT^ method.

### Plasmid construction and lentivirus infection

Previously, we constructed the recombinant PCDH-EF1-MCS-T2A-Puro-CRKL expression vector to overexpress CRKL in HCC cell lines [26]. To overexpress ETV6 in HCC cell lines, the full-length coding sequence of *ETV6* was first amplified by RT-PCR using forward primer 5′- TAGCTAGCGCCACCATGTCTGAGACTCCTGCTC-3′ and reverse primer 5′- GCCGCGCTTCGAATCAGCATTCATCTTCTTGG-3′, then inserted into the *Nhe* I and *BstB* I sites of a PCDH-EF1-MCS-T2A-Puro vector. The recombinant expression vector PCDH-EF1-MCS-T2A-Puro-ETV6 was used for overexpressing ETV6 in cells, the empty vector PCDH-EF1-MCS-T2A-Puro was used as control.

Lentivirus packaging was performed according to the manufacturer′s instructions, the recombinant expression vectors of PCDH-EF1-MCS-T2A-Puro-CRKL, PCDH-EF1-MCS-T2A-Puro-ETV6, PCDH-EF1-MCS-T2A-Puro were separately mixed with the packaging plasmids psPAX2 and pMD2G, and transfected into 293 T cells using Lipofectamine™ 2000 (Invitrogen, USA) for 48 h. The virus particle supernatants were harvested by centrifugation at 4500 rpm for 10 min and filtered with a 0.22 μm microporous membrane. Then, 1 × 10^5^ HepG2, HCCLM3 and HuH7 cells were infected with lentivirus in 6-well plates containing 8 μg/ml polybrene (Solarbio, China) for 48 h in a humidified incubator at 37 °C with 5% CO_2_. The cells stably transfected with PCDH-EF1-MCS-T2A-Puro-CRKL, PCDH-EF1-MCS-T2A-Puro-ETV6 and PCDH-EF1-MCS-T2A-Puro were screened against 0.5 μg/ml puromycin for 21 d at 37 °C with 5% CO2.

### siRNA design and transient transfection

For CRKL and ETV6 knockdown, targeting siRNAs (small interfering RNA) were designed according to *CRKL* sequence (Genbank: NM_005207.3, siCRKL: 5′-GTCACAAGGATGAATATAA-3′) and *ETV6* sequence (Genbank: NM_001987.4; siETV6–1: 5′-CAATATAGGTCTCAGAAATCC-3′; siETV6–2: 5′-GCATTAAGCAGGAACGAAT-3′; siETV6–3: 5′-GGGATTACGTCTATCAGTT-3′) using Invitrogen, siDirect and Whitehead software. Meanwhile, one siRNA with non-targeting sequence 5′-TTCTCCGAACGTGTCACGT-3′ was designed as a negative control (NC). One day before transfection, 3 × 10^5^ cells/well in 2 ml DMEM supplemented with 15% FBS were seeded into a 6-well plate, and the siRNA mixtures (2 μl siETV6–1 + 2 μl siETV6–2 + 2 μl siETV6–3) were transfected into HepG2, HCCLM3 and HuH7 cells using 5 μl Lipofectamine™ 2000 (Invitrogen, USA) according to the manufacturer’s instructions for 48 h at 37 °C with 5% CO_2_, respectively.

### In vitro cell migration and invasion assays

The effect of CRKL and ETV6 deregulations on the migration and invasion abilities of HepG2, HCCLM3 and HuH7 cells were examined using the Boyden transwell chamber assay. Briefly, 1 × 10^4^ cells in 200 μl serum-free DMEM were seeded onto the upper compartment of transwell with 8 μm pore size polycarbonate filters (Corning, USA). The chambers were then placed into 24-well plates containing 600 μl DMEM with 20% FBS and incubated for 24 h at 37 °C with 5% CO_2_. For invasion assay, the inserts were first coated with 50 μl 2.5% ECM gel (Sigma, USA) in DMEM, and incubated at 37 °C for 1 h. 1 × 10^4^ cells in 200 μl serum-free DMEM were seeded onto the upper compartment of the transwell. The chambers were then placed into 24-well plates containing 600 μl DMEM with 20% FBS and incubated for 24 h at 37 °C with 5% CO_2_. The non-migrated and non-invaded cells on the upper surface of the insert were removed by swabbing, the migrated and invaded cells onto the lower surface were fixed with methanol (AR, Sigma, US) for 30 min, stained with 0.1% crystal violet for 40 min, washed with phosphate buffered solution (PBS), counted using an upright light microscope (Olympus, Japan) with 100× magnification. Five random field views were counted and averaged.

### F-actin cytoskeleton staining assay

The TRITC (tetramethyl rhodamin isothiocyanate)-Phalloidin staining assay was performed to investigate the influence of CRKL and ETV6 on the cytoskeleton structure of HepG2, HCCLM3 and HuH7 cells. The 0.17 mm thick round cover glasses of 25 mm diameter were placed into 6-well plates, then 1 × 10^5^ cells from each group were seeded into the cover glasses and cultured for 24 h at 37 °C in humidified incubator with 5% CO_2_. The cover glasses were then twice washed with 1 ml PBS, fixed in 4% paraformaldehyde for 10 min at RT, washed 3 times with PBS, permeabilized with acetone, and continuously incubated in 100 μl TRITC-Phalloidin (400 nM) containing 1% bovine serum albumin (BSA) for 30 min in the dark at RT. The cover glasses were washed with PBS for 3 times and counterstained in 100 μl 50 nM Hoechst 33342 staining solution (Sigma, USA) for cell nucleus. Images were immediately taken using a fluorescence microscope (Olympus, Japan) at five randomly selected visual fields of 1000× within 1 h.

### Co-immunoprecipitation assay

Co-immunoprecipitation (Co-IP) assay was performed to determine the interaction between CRKL and ETV6. Total protein was extracted from 5 × 10^6^ HCCLM3-PCDH-CRKL or HCCLM3-PCDH-NC cells using RIPA buffer, then 500 μg protein was incubated with 2 μl anti-CRKL (Santa Cruz Biotechnology, USA), 2 μl anti-ETV6 (abcam, USA), or 1 μl nonspecific anti-rabbit IgG (Santa Cruz Biotechnology, USA) for 1 h at 4 °C. The immune complexes were pulled down by 20 μl protein A/G agarose beads (Santa Cruz Biotechnology, USA) at 4 °C overnight. Then the beads were washed 3 times with 500 μl RIPA buffer at RT, and after centrifugation with 12,000 rpm for 15 min at 4 °C, the beads were mixed with 4 μl 5 × SDS-PAGE loading buffer and 16 μl RIPA and boiled for 10 min. Then the immunoprecipitated proteins were separated by 10% SDS-PAGE and analyzed by WB.

### Chromatin immunoprecipitation (ChIP) assay

ChIP assay was performed to investigate the interaction between ETV6 and miR-429 using an EZ-ChIP™ Chromatin Immunoprecipitation Kit (Millipore, USA). Briefly, 2 × 10^7^ HCCLM3 cells were cross-linked with 550 μl 37% formaldehyde for 10 min at RT and treated with 2 ml 10 × glycine for 5 min at RT, then the cells were washed with 2 ml PBS containing 10 μl protease inhibitor cocktail II, scraped and lysed with 1 ml SDS lysis buffer containing 5 μl protease inhibitor cocktail II. Then the lysates were sonicated on ice for 6 × 15 s to shear the DNA and the debris was removed by centrifugation at 12000 rpm for 10 min at 4 °C. A small part of the lysate was used as DNA input control, the remaining part was diluted 10-fold with dilution buffer and incubated with 4 μl anti-ETV6 (Abcam, USA) or 1 μl anti-RNA polymerase II, 1 μl normal mouse IgG, 4 μl anti-rabbit IgG at 4 °C overnight. Immunoprecipitated complexes were collected using 60 μl protein A/G agarose beads at 4 °C for 1 h. After centrifugation at 5000 rpm for 1 min at 4 °C, the precipitates were washed with 1 ml washing buffer at 4 °C for 3 × 5 min, and eluted with elution buffer (20% SDS and 1 M NaHCO_3_) for 15 min at RT. Cross-linking of protein-DNA complexes was reversed with 8 μl 5 M NaCl at 65 °C overnight, followed by treatment with 1 μl RNase A at 37 °C for 30 min, and 4 μl 0.5 M EDTA, 8 μl 1 M Tris-HCl, 1 μl Proteinase K at 45 °C for 2 h. Then, DNA was extracted using a spin column according to the manufacturer’s instructions. The DNA was subjected to PCR to amplify the 262 bp promoter region of miR-429 using specifically the designed primers, F: 5′-ACCTCGCCACCGCCTCCCATTGTCCCGTCG-3′ and R: 5′-TGCCAGGCCCGGGTGGGTGTGAACCGGCTTC-3′.

### Data processing and statistical analysis

Statistical analyses were performed using GraphPad Prism 5.0 software. The data were presented as mean ± SD of at least three independent experiments. The statistical differences between groups were evaluated by Student’s t-test analysis. As most of the distributions deviated from the normal Gaussian distribution, non-parametric tests were used for further analyses. The correlations between the expression of ETV6, CRKL and miR-429 were analyzed using the Spearman’s rank correlation coefficient. Differences with *P* ≤ 0.05 are statistically significant.

## Results

### Expression patterns and the correlations of ETV6, CRKL and miR-429 in hepatocarcinoma patients’ tumorous tissues and HCC cell lines

**Fig. 1:**
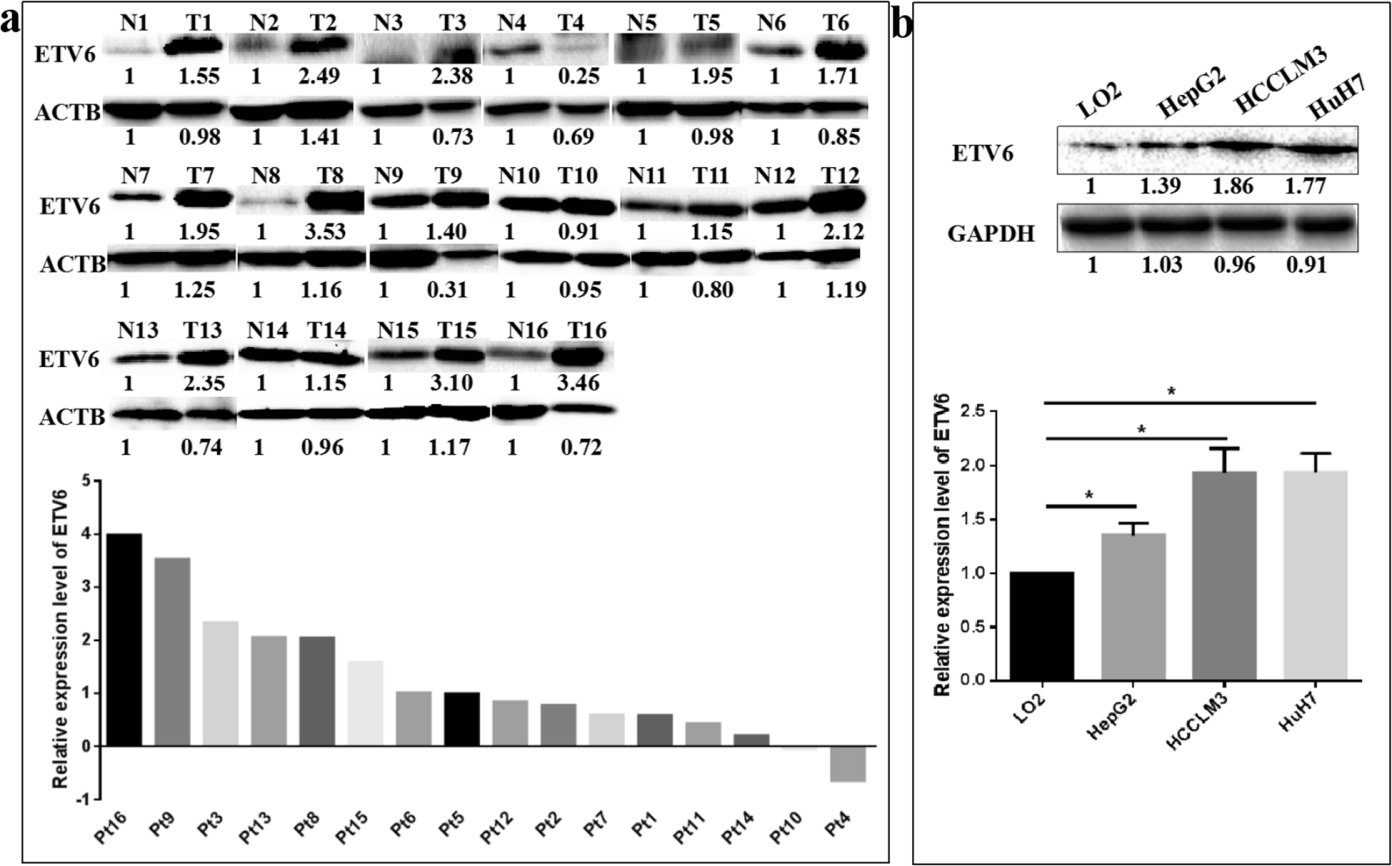
The expression patterns of ETV6 in hepatocarcinoma patients’ tumorous tissues and HCC cells. **a** WB measured the protein expression level of ETV6 in hepatocarcinoma patients’ tumorous tissues and corresponding non-tumor liver tissues. ETV6 was overexpressed in hepatocarcinoma patients’ tumorous tissues. **b** WB measured the protein expression level of ETV6 in HCC HepG2, HCCLM3 HuH7 cells and human normal liver LO2 cells. ETV6 was overexpressed in HCC cells

To investigate the potential role of ETV6, CRKL and miR-429 in hepatocarcinoma, we detected the expression levels of ETV6, CRKL and miR-429 in HCC tissues and cells. WB results showed that compared with non-tumor liver tissues, ETV6 expression in tumorous tissues of hepatocarcinoma patients was increased by 127.6% (*P* = 0.0005, Fig. 1a). Meanwhile, ETV6 was expressed in all the tested cell lines and a comparatively low expression was shown in normal liver LO2 cells compared with the hepatocarcinoma cells. Compared with normal liver LO2 cells, the expression levels of ETV6 in HepG2, HCCLM3 and HuH7 cells were increased by 34.9% (*P* = 0.0499, Fig. 1b), 93.3% (*P* = 0.0284, Fig. 1b) and 94.0% (*P* = 0.0166, Fig. 1b). Our results suggested that ETV6 displayed a comparatively high expression in hepatocarcinoma tissues and cells, and that it might be a potential biomarker for hepatocarcinoma progression.

**Fig. 2:**
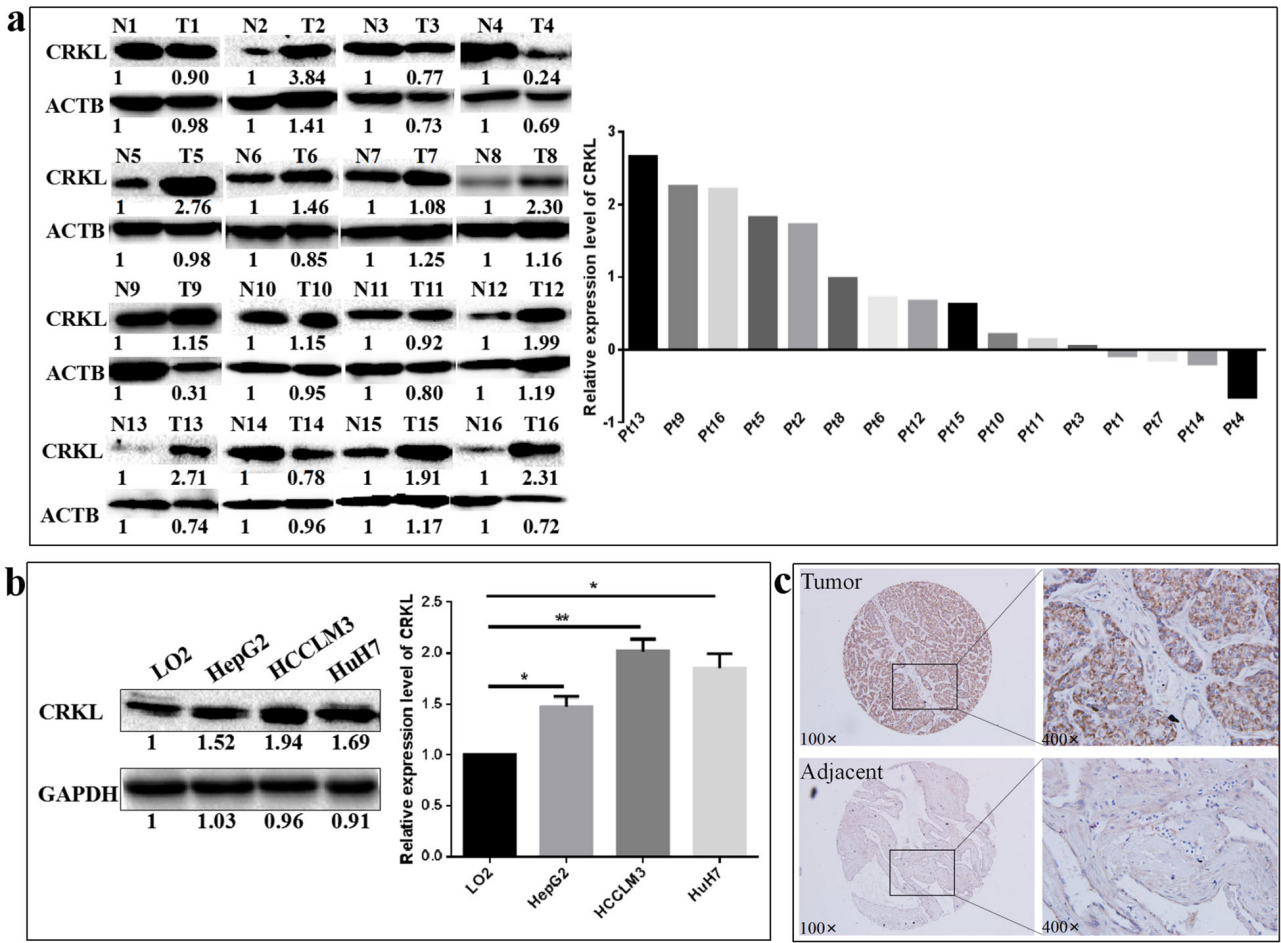
The expression patterns of CRKL in hepatocarcinoma patients’ tumorous tissues and HCC cells. **a** WB measured the protein expression level of CRKL in hepatocarcinoma patients’ tumorous tissues and corresponding non-tumor liver tissues. CRKL was overexpressed in hepatocarcinoma patients’ tumorous tissues. **b** WB measured the protein expression level of ETV6 in HCC HepG2, HCCLM3 HuH7 cells and human normal liver LO2 cells. CRKL was overexpressed in HCC cells. **c** IHC measured the protein expression level of CRKL in 48 paired tumorous and adjacent normal tissues from HCC patients

CRKL was also upregulated in hepatocarcinoma patients’ tumorous tissues and hepatocarcinoma cell lines. As shown in Fig. 2a, WB assay showed CRKL expression was upregulated by 79.6% (*P* = 0.0041) in the tumorous tissues from hepatocarcinoma patients. Meanwhile, CRKL expression levels were increased by 47.2% (*P* = 0.0221), 101.7% (*P* = 0.0068) and 85.5% (*P* = 0.0134) in HepG2, HCCLM3 and HuH7 cells compared with LO2 cell (Fig. 2b). A tissue microarray composed of 48 paired tumorous and adjacent normal tissues from HCC patients was further employed to address the expression alteration of CRKL by IHC. Among the 48 cases with hepatocarcinoma, 28 showed strong, 15 showed moderate, 5 showed weak and 0 showed negative CRKL expression. The positive immunostaining rate of CRKL in hepatocarcinoma tissues was 2.15-fold compared to adjacent normal tissues (Table 1). The representative examples for CRKL expression in hepatocarcinoma tissues and adjacent normal tissues were shown in Fig. 2c. These results demonstrated the overexpression of CRKL in hepatocarcinoma and indicated the likelihood of its high expression being involved in the development and progression of hepatocarcinoma.

**Table 1:** CRKL was overexpressed in tumor tissues from 48 HCC patients

Group	Total	CRKL				*P*
		–	+	++	+++	
Tumor tissues	48	0	5	15	28	0.008
Adjacent tissues	48	0	5	30	13	

**Fig. 3:**
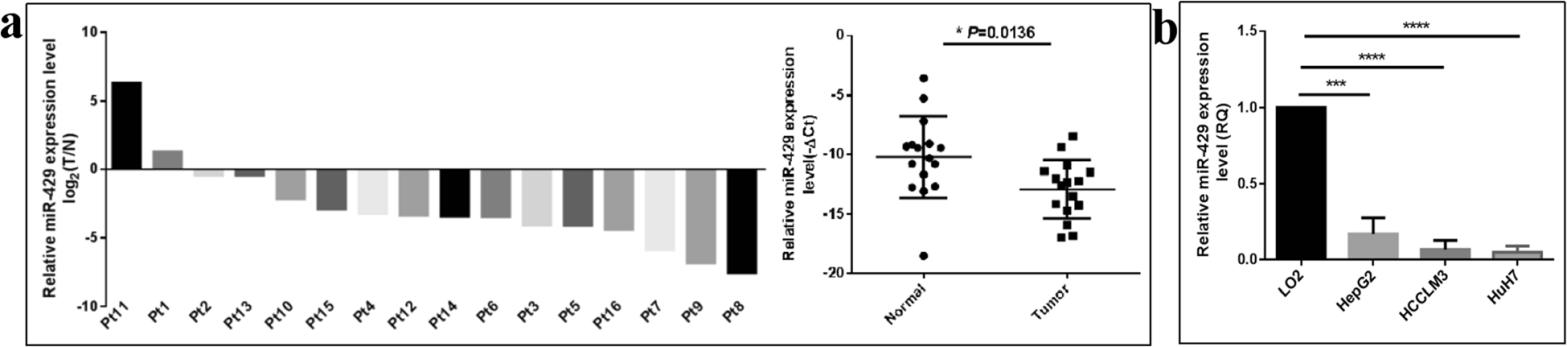
The expression patterns of miR-429 in hepatocarcinoma patients’ tumorous tissues and HCC cells. **a** qRT-PCR measured the expression level of miR-429 in in hepatocarcinoma patients’ tumorous tissues and corresponding non-tumor liver tissues. miR-429 was poorly expressed in hepatocarcinoma patients’ tumorous tissues. **b** qRT-PCR measured the expression level of miR-429 in HepG2, HCCLM3, HuH7 and LO2 cells. miR-429 was poorly expressed in HCC cells

Furthermore, we measured that miR-429 was significantly down-regulated in the tumorous tissues of hepatocarcinoma patients and in hepatocarcinoma cell lines. As shown in Fig. 3a, qRT-PCR assays showed that the miR-429 expression level was downregulated by 27.5% (*P* = 0.0136) in the tumorous tissues from hepatocarcinoma patients. Furthermore, miR-429 was expressed in all the tested cell lines and a comparatively high expression was shown in normal liver LO2 cells compared to hepatocarcinoma cells. miR-429 expression levels were decreased by 83.0% (*P* = 0.0002), 93.3% (*P* < 0.0001) and 95.1% (*P* < 0.0001) in HepG2, HCCLM3 and HuH7 cells compared with LO2 cell (Fig. 3b). Taken together, our results indicated that ETV6, CRKL might act as tumor promoters and miR-429 as a tumor suppressor involved in the development and progression of HCC.

**Fig. 4:**
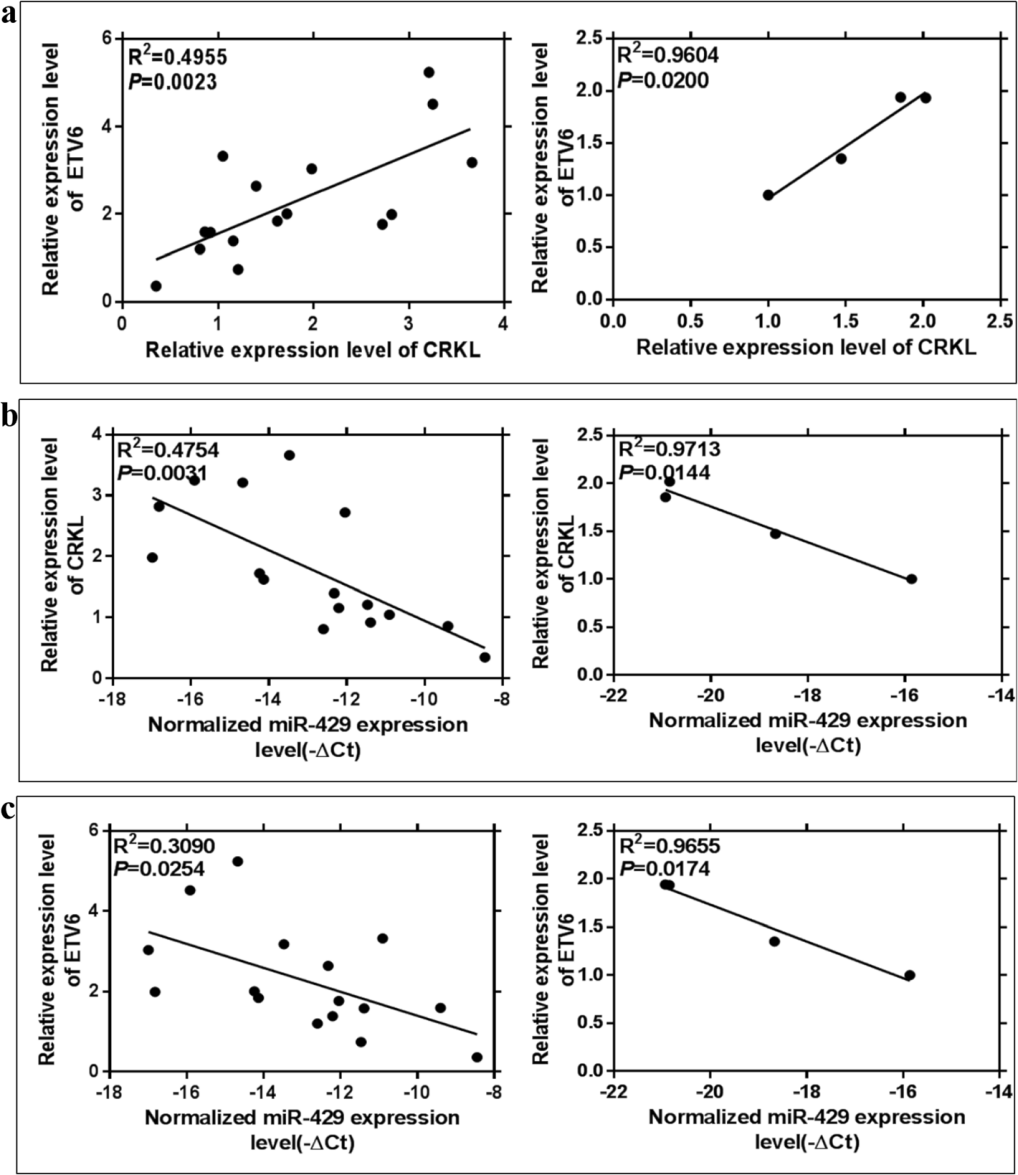
The correlations of ETV6, CRKL and miR-429 expression level changes in hepatocarcinoma. **a** ETV6 expression level was positively correlated with CRKL expression in both hepatocarcinoma patients’ tumorous tissues and in hepatocarcinoma cells. **b** CRKL expression level was negatively correlated with miR-429 expression in both hepatocarcinoma patients’ tumorous tissues and in hepatocarcinoma cells. **c** ETV6 expression level was negatively correlated with miR-429 expression in both hepatocarcinoma patients’ tumorous tissues and in hepatocarcinoma cells

We further analyzed the inter-correlations of ETV6, CRKL and miR-429 expression level changes in tumorous tissues of hepatocarcinoma patients and in hepatocarcinoma cells. As shown in Fig. 4a, the upregulations of ETV6 and CRKL were positively correlated in both hepatocarcinoma patients’ tumorous tissues (*R*^2^ = 0.4955, *P* = 0.0136, Fig. 4a) and in hepatocarcinoma cells (*R*^2^ = 0.9604, *P* = 0.0200, Fig. 4a). The CRKL expression level change was negatively correlated with miR-429 in both hepatocarcinoma patients’ tumorous tissues (*R*^2^ = 0.4754, *P* = 0.0031, Fig. 4b) and in hepatocarcinoma cells (*R*^2^ = 0.9713, *P* = 0.0144, Fig. 4b). A negative correlation was also established for ETV6 upregulation with miR-429 downregulation in both hepatocarcinoma patients’ tumorous tissues (*R*^2^ = 0.3090, *P* = 0.0254, Fig. 4c) and in hepatocarcinoma cells (*R*^2^ = 0.9655, *P* = 0.0174, Fig. 4c). Our results demonstrated that ETV6 expression is positively correlated with CRKL expression, while miR-429 expression is simultaneously negatively correlated with CRKL and ETV6 expression, and the dysexpressions of ETV6, CRKL and miR-429 were closely correlated in affecting hepatocarcinoma malignancy.

### ETV6 deregulation affects the in vitro migration, invasion and cytoskeleton arrangement of HCC cells

**Fig. 5:**
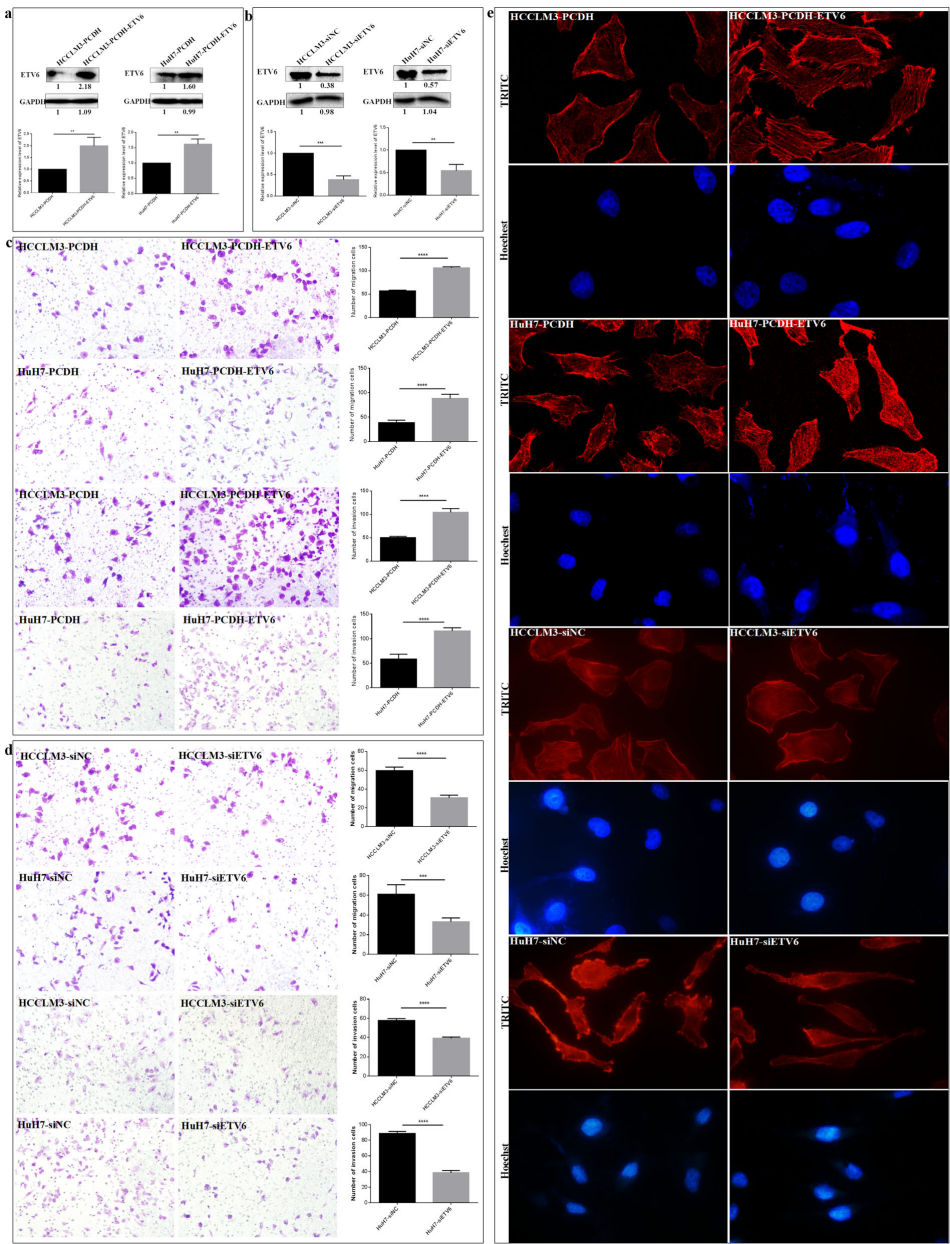
ETV6 dysexpression affects the in vitro migration, invasion and F-actin cytoskeleton expression and arrangement of HCC cells. **a** ETV6 were over-expressed in HCCLM3 and HuH7 cells stably transfected with PCDH-EF1-MCS-T2A-Puro-ETV6 vector by Lentivirus infection against puromycin screening. Relative ETV6 protein expression levels were determined by WB using GAPDH as the internal reference. **b** ETV6 were downregulated in HCCLM3 and HuH7 cells by siETV6 transient transfection interference. Relative protein levels were determined by WB using GAPDH as the internal reference. Transwell assays indicated ETV6 overexpression **c** enhanced and ETV6 knockdown **d** decreased the in vitro migration and invasion capacities of HCCLM3 and HuH7 cells. **e** FITC-phalloidin assays indicated ETV6 overexpression or downregulation increased or decreased cytoskeleton F-actin protein expression level and filament arrangement of HCCLM3 and HuH7 cells

To confirm its tumor promotion effect in HCC, we measured both the overexpression and knockdown of ETV6 on the migration and invasion capacities of HCCLM3 and HuH7 cells. First, we successfully overexpressed ETV6 in HCCLM3 and HuH7 cells stably transfected with PCDH-EF1-MCS-T2A-Puro-ETV6 recombinant vector by Lentivirus infection against puromycin screening. In comparison with HCCLM3-PCDH and HuH7-PCDH cells, ETV6 protein expression levels were increased by 99.7% (*P =* 0.0084) and 61.7% (*P* = 0.003) in HCCLM3-PCDH-ETV6 and HuH7-PCDH-ETV6 cells, respectively (Fig. 5a). The establishment of HCCLM3-PCDH-ETV6 and HuH7-PCDH-ETV6 cells with stable ETV6 overexpression provides a control study for the upregulation effect of ETV6 on the malignant behaviours HCCLM3 and HuH7 cells. Meanwhile, we knockdown ETV6 expression in HCCLM3 and HuH7 cells using siETV6 transient transfection. In comparison with HCCLM3-siNC and HuH7-siNC cells, ETV6 protein expression levels decreased by 61.3% (*P =* 0.0002) and 45.3% (*P* = 0.0044) (Fig. 5b) in HCCLM3-siETV6 and HuH7-siETV6 cells, which provides a control study for investigating ETV6 suppression on the malignant behaviours of hepatocarcinoma cells.

ETV6 dysexpression apparently affects the in vitro migration and invasion capacities of HCCLM3 and HuH7 cells. ETV6 expression level was positively correlated with the migration and invasion abilities of HCCLM3 and HuH7 cells. ETV6 overexpression enhanced the migration and invasion abilities of HCCLM3 and HuH7 (Fig. 5c). The numbers of migrated HCCLM3-PCDH-ETV6 (106.2 ± 1.1) and HuH7-PCDH-ETV6 (88.4 ± 3.8) cells increased by 86.0% (*P* = 0.0001) and 125.6% (*P* = 0.0001) than those of HCCLM3-PCDH (57.0 ± 0.7) and HuH7-PCDH cells (38.8 ± 2.3). The numbers of invaded HCCLM3-PCDH-ETV6 (104.6 ± 3.4) and HuH7-PCDH-ETV6 (116.2 ± 2.9) cells increased by 110.0% (*P* = 0.0001) and 96.6% (*P* = 0.0003) than those of HCCLM3-PCDH (50.4 ± 1.2) and HuH7-PCDH cells (58.8 ± 4.4). Consistently, ETV6 downregulation inhibited the migration and invasion abilities of HCCLM3 and HuH7 cells (Fig. 5d). The numbers of migrated HCCLM3-siETV6 (30.8 ± 1.2) and HuH7-siETV6 (33.2 ± 1.7) cells decreased by 48.3% (*P* = 0.0001) and 57.3% (*P* = 0.0003) than HCCLM3-siNC (59.8 ± 1.7) and HuH7-siNC cells (61.0 ± 4.3). The numbers of invaded HCCLM3-siETV6 (39.2 ± 0.6) and HuH7-siETV6 (38.4 ± 1.2) cells decreased by 32.8% (*P* = 0.0001) and 45.9% (*P* = 0.0003) than HCCLM3-siNC (57.8 ± 0.9) and HuH7-siNC cells (88.8 ± 1.2). Clearly, ETV6 significantly affected the in vitro migration and invasion abilities of HCCLM3 and HuH7 cells. Our results indicated that ETV6 acts as a tumor promoter in hepatocarcinoma by promoting the aggressiveness of tumor cells.

It is well established that cytoskeleton elements are closely related to cell movement. The changes of F-actin microfilament organization in HCCLM3 and HuH7 cells after ETV6 deregulation was investigated by fluorescence microscopy using FITC-labeled phalloidin. ETV6 deregulation significantly affected F-actin cytoskeleton protein expression of HCCLM3 and HuH7 cells. As shown in Fig. 5e, ETV6 overexpression resulted in an obvious increase of F-actin microfilament. HCCLM3-PCDH-ETV6 and HuH7-PCDH-ETV6 cells showed a regular array of actin filaments present along the cells that evenly radiated to the cell membrane from the nucleus. While, ETV6 knockdown decreased F-actin cytoskeleton protein expression and microfilament arrangement of HCCLM3 and HuH7 cells, HCCLM3-siETV6 and HuH7-siETV6 exhibited a reduced amount of F-actin and a disorganization of actin filaments. Our results showed ETV6 acts as a promoter for HCCLM3 and HuH7 cells migration and invasion by destructing its F-actin cytoskeleton. Taken together, ETV6 deregulation affected the cytoskeleton F-actin expression and arrangement of HCCLM3 and HuH7 cells, which leads to its migration and invasion potentials.

### CRKL deregulation affects the in vitro migration, invasion and cytoskeleton arrangement of HCC cells

**Fig. 6:**
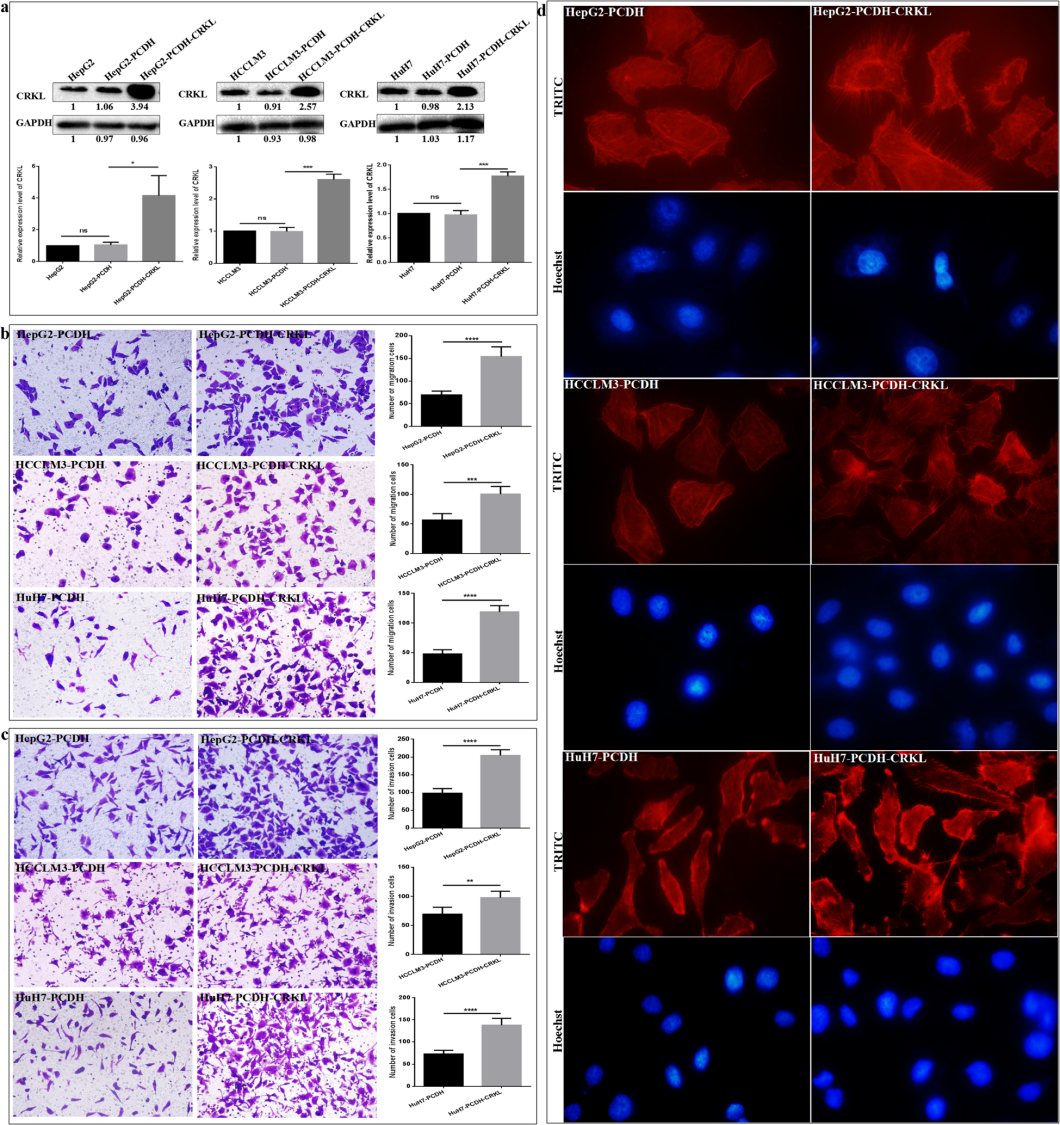
CRKL overexpression affects the in vitro migration, invasion and F-actin cytoskeleton expression and arrangement of HCC cells. **a** CRKL were over-expressed in HepG2, HCCLM3 and HuH7 cells. PCDH-EF1-MCS-T2A-Puro-CRKL or PCDH-EF1-MCS-T2A-Puro were stably transfected into HepG2, HCCLM3 and HuH7 cells, screened against 0.5 μg/ml puromycin. Relative CRKL protein expression levels were determined by WB using GAPDH as internal reference. **b** and **c** Transwell assays showed that CRKL overexpression promoted the in vitro migration and invasion abilities of HepG2, HCCLM3 and HuH7 cells. **d** FITC-phalloidin assays showed that CRKL overexpression increased cytoskeleton F-actin protein expression and filament arrangement of HepG2, HCCLM3 and HuH7 cells

Previously, we obtained monoclonal HepG2-shCRKL cells with stable CRKL knockdown and transiently transfected HepG2 cells with a PCDH-EF1-MCS-T2A-Puro-CRKL expression plasmid for overexpressing CRKL to investigate the effects of CRKL deregulation on migration and invasion abilities of HepG2 cells. We found CRKL expression levels were positively related to the in vitro migration and invasion abilities of hepatocarcinoma HepG2 cells [26]. In the current study, we further measured the effects of CRKL expression level change on the migration, invasion and cytoskeleton of HCC cells by stably up-regulating CRKL in HepG2, HCCLM3 and HuH7 cells. The stable transfection with PCDH-EF1-MCS-T2A-Puro-CRKL vector by Lentivirus infection, combined with puromycin screening led to the stable overexpression of CRKL in HepG2, HCCLM3 and HuH7 cells. No protein level change differences were observed for CRKL between HepG2 and HepG2-PCDH cells, HCCLM3 and HCCLM3-PCDH, or HuH7 and HuH7-PCDH cells (Fig. 6a). In comparison with empty vector transfected HepG2-PCDH, HCCLM3-PCDH and HuH7-PCDH cells, CRKL protein levels were increased by 310.1% (*P =* 0.0106), 162.0% (*P* = 0.0001) and 82.1% (*P* = 0.0004) in HepG2-PCDH-CRKL, HCCLM3-PCDH-CRKL and HuH7-PCDH-CRKL cells, respectively, (Fig. 6a). The establishment of HepG2-PCDH-CRKL, HCCLM3-PCDH-CRKL and HuH7-PCDH-CRKL cells with stable CRKL overexpression provides a control study for the upregulation effect of CRKL on the malignant behaviours of HepG2, HCCLM3 and HuH7 cells.

CRKL levels were positively correlated with the migration and invasion capacities of HepG2, HCCLM3 and HuH7 cells in vitro. CRKL overexpression promoted the migration and invasion abilities of HepG2, HCCLM3 and HuH7 cells. As shown in Fig. 6b, the numbers of migrated HepG2-PCDH-CRKL, HCCLM3-PCDH-CRKL and HuH7-PCDH-CRKL cells were measured as 150.4 ± 9.6, 100.4 ± 4.8, 118.8 ± 4.8, which were increased by 122.5% (*P* < 0.0001), 77.4% (*P* = 0.0004), 148.5% (*P* < 0.0001) compared with HepG2-PCDH (69.2 ± 4.8), HCCLM3-PCDH (56.6 ± 4.8) and HuH7-PCDH (47.8 ± 3.2) cells, respectively. The numbers of invaded HepG2-PCDH-CRKL (204.4 ± 7.2), HCCLM3-PCDH-CRKL (100.4 ± 5.8) and HuH7-PCDH-CRKL (137.8 ± 6.9) cells increased by 108.6% (*P* < 0.0001), 41.2% (*P* = 0.0004), 88.3% (*P* < 0.0001) compared with HepG2-PCDH (98.0 ± 5.0), HCCLM3-PCDH (69.4 ± 5.3) and HuH7-PCDH (73.2 ± 3.7) cells, respectively (Fig. 6c). Clearly, CRKL promoted the in vitro migration and invasion abilities of HepG2, HCCLM3 and HuH7 cells. Our results indicated that CRKL acts as a tumor promoter in hepatocarcinoma by promoting tumor cell’s aggressiveness.

Furthermore, the TRITC-Phalloidin cytoskeleton staining assay indicated that CRKL overexpression resulted in an obviously increased intracellular microfilament of F-actin and spreading lamellipodia in HepG2, HCCLM3 and HuH7 cells (Fig. 6d). HepG2-PCDH-CRKL, HCCLM3-PCDH-CRKL and HuH7-PCDH-CRKL cells showed more abundant and ordered microfilaments, but the control group cells showed fewer and disordered actin. Our results showed CRKL acting as a promoter for HCCLM3 and HuH7 cells migration and invasion by destructing the F-actin cytoskeleton. Taken together, CRKL overexpression promoted F-actin cytoskeleton protein expression and arrangement of HepG2, HCCLM3 and HuH7 cells, leading to its increased migration and invasion potential.

### ETV6 directly binds to CRKL to positively regulate its expression

**Fig. 7:**
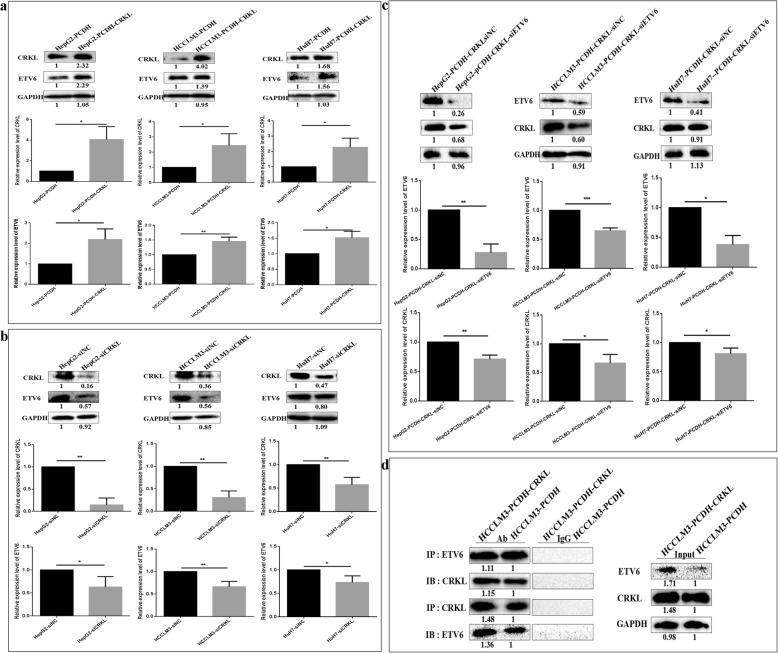
ETV6 directly binds to CRKL and positively regulates its expression. **a** CRKL overexpression increased ETV6 expression levels in HepG2, HCCLM3 and HuH7 cells. **b** CRKL knockdown decreased ETV6 expression levels in HepG2, HCCLM3 and HuH7 cells. **c** ETV6 knockdown decreased CRKL expression levels in CRKL-overexpressing HepG2, HCCLM3 and HuH7 cells. **d** Co-IP assays detected the direct interaction between ETV6 and CRKL

After, we observed a significant positive correlation between the expression levels of ETV6 and CRKL in hepatocarcinoma tumor tissues and hepatocarcinoma cell lines, then we investigated the mutual influence between the dysexpression of ETV6 and CRKL. CRKL overexpression increased endogenous ETV6 expression levels (Fig. 7a). In comparison with HepG2-PCDH, HCCLM3-PCDH and HuH7-PCDH cells, ETV6 expression levels were increased by 118.5% (*P* = 0.0372), 45.2% (*P* = 0.0075), 51.5% (*P* = 0.0338) in HepG2-PCDH-CRKL, HCCLM3-PCDH-CRKL and HuH7-PCDH-CRKL cells. CRKL knockdown consistently resulted in decreased ETV6 expression by 37.0% (*P* = 0.0253), 33.9% (*P* = 0.0082), 26.7% (*P* = 0.0295) in HepG2, HCCLM3 and HuH7 cells (Fig. 7b). Moreover, the effect of ETV6 expression level changes on CRKL were also investigated by transiently transfecting si-ETV6 in HepG2, HCCLM3 and HuH7 cells with CRKL overexpression. ETV6 downregulation decreased exogenous CRKL expression levels (Fig. 7c). Compared with HepG2-PCDH-CRKL-siNC, HCCLM3-PCDH-CRKL-siNC, HuH7-PCDH-CRKL-siNC cells, CRKL protein expression levels were decreased by 28.9% (*P* = 0.0046), 33.7% (*P* = 0.0224), 19.1% (*P* = 0.0181) in HepG2-PCDH-CRKL-siETV6, HCCLM3-PCDH-CRKL-siETV6 and HuH7-PCDH-CRKL-siETV6 cells. The results demonstrated ETV6 expression was positively correlated with CRKL expression in hepatocarcinoma cells.

Tel-ABL fusion protein may form complexes with CRKL [10], and thus we proposed that ETV6 and CRKL might regulate each other directly by forming a complex. Co-IP experiment confirmed their direct interaction in HCCLM3 cells. The extracted proteins from HCCLM3-PCDH-CRKL and HCCLM3-PCDH cells were immunoprecipitated using antibody against CRKL, ETV6, and the control non-specific IgG, respectively, and the eluted proteins were detected by WB using antibody against ETV6, CRKL, the acetylated antibody, respectively. Our results clearly revealed that a CRKL protein band or ETV6 protein band appeared in the immunoprecipitated complexes with antibodies against ETV6 or CRKL, but not the non-specific IgG, meanwhile, the amount of CRKL-ETV6 complexes obtained was greater in HCCLM3-PCDH-CRKL cells than in HCCLM3-PCDH cells (Fig. 7d). Our results indicated that ETV6 directly binds to CRKL to positively regulate its expression.

### ETV6 negatively regulates miR-429 by directly binding to its promoter region

**Fig. 8:**
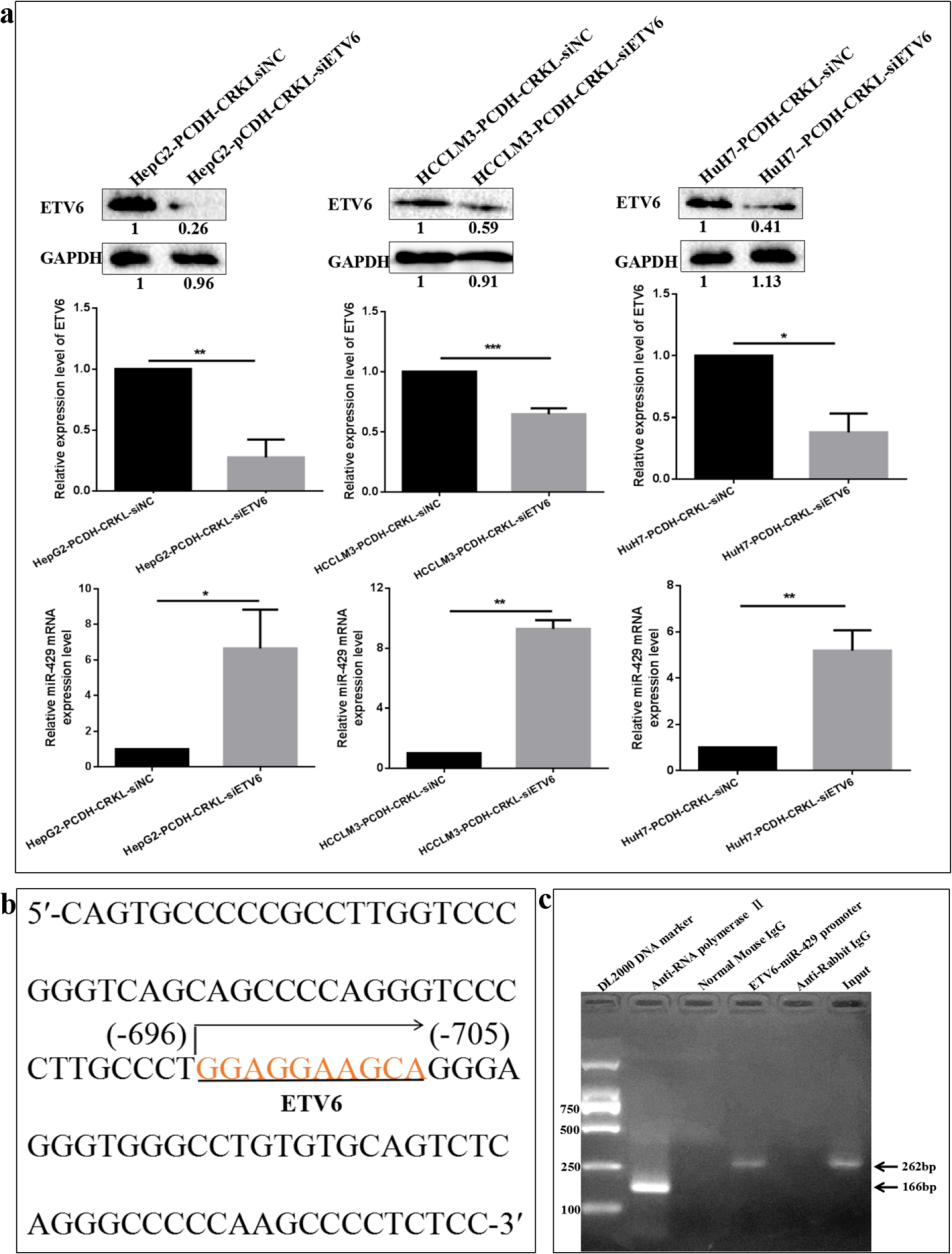
ETV6 negatively regulates miR-429 by directly binding to its promoter region. **a** ETV6 downregulation increased miR-429 expression level in CRKL-overexpression HepG2, HCCLM3 and HuH7 cells. **b** Putative binding site for ETV6 protein at promoter region of miR-429. **c** ChIP assay of the binding of ETV6 to the promoter region of miR-429. Anti-RNA polymerase II was used as a positive control for binding to the GAPDH promoter region, normal mouse IgG was as a negative control for binding to the GAPDH promoter region, anti-ETV6 was used for experimental group on binding to the miR-429 promoter region, anti-rabbit IgG was used as a control. PCR products were analyzed by 1% agarose gel

We observed a significant negative correlation between the expression level of ETV6 and miR-429 in hepatocarcinoma tumor tissues and hepatocarcinoma cell lines. To further confirm the negative correlation between ETV6 and miR-429 expression levels, we detected the effect of ETV6 knockdown on the expression level of miR-429 by transfecting si-ETV6 in CRKL-overexpression HepG2, HCCLM3 and HuH7 cells. ETV6 downregulation resulted in increased expression levels of miR-429 (Fig. 8a). In comparison with HepG2-PCDH-CRKL-siNC, HCCLM3-PCDH-CRKL-siNC and HuH7-PCDH-CRKL-siNC cells, miR-429 expression levels were increased by 5.9-fold (*P* = 0.0091), 8.4-fold (*P* = 0.0050) and 4.2-fold (*P* = 0.0031) in HepG2-PCDH-CRKL-siETV6, HCCLM3-PCDH-CRKL-siETV6 and HuH7-PCDH-CRKL-siETV6 cells. Our results further demonstrated that ETV6 expression was negatively correlated with miR-429 expression.

Bioinformatics analysis software JASPAR (http://jaspar2016.genereg.net/cgi-bin/jaspar_db.pl?rm=browse&db=core&tax_group=vertebrates) indicated that ETV6 protein potentially binds to the DNA promoter region at the 696–705 site of miR-429 (Fig. 8b). A ChIP experiment was performed to verify the binding interaction using HCCLM3 cells. The miR-429 promoter region in the precipitated chromatin was amplified by PCR. As shown in Fig. 8c, the 262 bp PCR product containing the binding site for ETV6 protein at the promoter region of miR-429 was specifically detected in anti-ETV6 ChIP and in the input ChIP, but not in anti-rabbit IgG ChIP. Meanwhile, the 166 bp PCR product of GAPDH promoter was observed in anti-RNA polymerase II ChIP, but not in the normal Mouse IgG ChIP. The above results indicated the direct binding of ETV6 to the promoter region of miR-429 mediates its functionality.

### miR-429 negatively regulates CRKL expression by selectively targeting its 3′-UTR

**Fig. 9:**
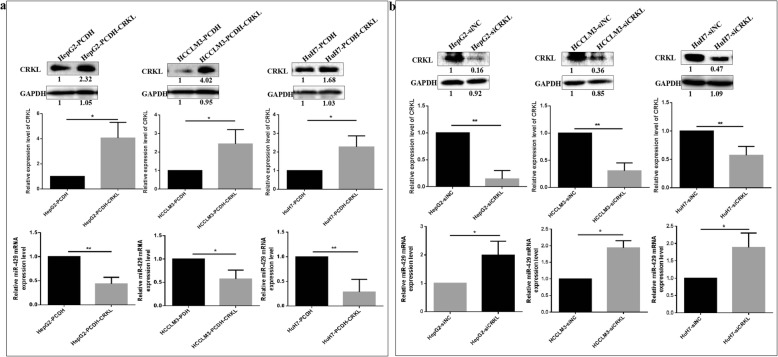
miR-429 negatively regulates CRKL expression by selectively targeting its 3′-UTR. **a** CRKL overexpression decreased miR-429 expression levels in HepG2, HCCLM3 and HuH7 cells. **b** CRKL knockdown increased miR-429 expression levels in HepG2, HCCLM3 and HuH7 cells

We also observed a significant negative correlation between the expression levels of miR-429 and CRKL in hepatocarcinoma tumor tissues and hepatocarcinoma cell lines. To further confirm the negative correlation between miR-429 and CRKL, we detected the effect of CRKL deregulation on the expression levels of miR-429 in HepG2, HCCLM3 and HuH7 cells. CRKL overexpression decreased endogenous miR-429 expression level (Fig. 9a). In comparison with HepG2-PCDH, HCCLM3-PCDH and HuH7-PCDH cells, miR-429 expression levels were decreased by 54.1% (*P* = 0.0091), 41.5% (*P* = 0.0311), 73.1% (*P* = 0.0030) in HepG2-PCDH-CRKL, HCCLM3-PCDH-CRKL and HuH7-PCDH-CRKL cells. Consistently, CRKL knockdown resulted in increased endogenous miR-429 expression levels by 94.5% (*P* = 0.0100), 90.1% (*P* = 0.0462), 89.7% (*P* = 0.0218) in HepG2, HCCLM3 and HuH7 cells (Fig. 9b). Our results further demonstrated miR-429 expression was negatively correlated with CRKL expression in hepatocarcinoma cells. Meanwhile, we have previous reported that CRKL was a direct downstream target of miR-429 via direct binding to site 2 in its 3′-UTR by post-transcriptionally mediating its functionality, and miR-429 overexpression and suppression decreased and increased endogenous CRKL expression levels in HepG2 cells [26]. Our results indicated miR-429 negatively regulates CRKL expression by selectively targeting its 3′-UTR.

## Discussion

The current study has identified a novel ETV6-miR-429-CRKL signaling axis that played an important role in HCC, and provided a comprehensive mechanism for the tumorigenesis of HCC in whihc ETV6 binding of miR-429 positively regulates CRKL expression. The information gained from this research has important clinical implications for patients with HCC as well as other cancer types associated with elevated ETV6, CRKL expression and decreased miR-429 expression, and may also have clinical impact on other diseases with dysregulated expression of ETV6, CRKL and miR-429. In current work, we only collected 16 pairs of matched tumour tissues and corresponding non-tumor liver tissues from hepatocarcinoma patients, the sample size is relatively small, but our results are reliable, because we also detected the expression patterns and the correlations of ETV6, CRKL and miR-429 in clear cell renal cell carcinoma (ccRCC) patients’ tumorous tissues and matched paracancerous nontumoral renal tissues, and bone marrow (BM) of chronic myeloid leukemia (CML) patients and peripheral blood (PB) of normal samples, the expression patterns and the correlations of ETV6, CRKL and miR-429 in ccRCC and CML consistent with those in liver cancer (unpublished). Previously, we have detected the effect of ETV6 on proliferation ability of HCC cells, we found re-expression or silencing of ETV6 slightly affected the proliferation and colony formation abilities of HCCLM3 and HuH7 cells. Meanwhile, re-expression or silencing of miR-429 cannot affect HCC cells proliferation and colony formation abilities, and CRKL only deregulated to a certain degree, which can affect HCC cells proliferation ability [26]. Our results indicated ETV6-miR-429-CRKL regulatory circuitry mainly contributed to migration and invasion abilities of HCC cells. So, in current work, we mainly investigated the potential role of ETV6-miR-429-CRKL in metastasis of HCC.

ETV6 is a leukemia-associated gene located on 12p13, which has been shown to play a wide role in hematopoiesis and hematological malignancies [9, 37, 38]. ETV6 frequently forms fusion proteins by chromosomal translocations with partner genes. At present 48 chromosomal bands have been identified to be involved in ETV6 translocations and 30 ETV6 partner genes have been molecularly characterized [11, 39]. Recently, ETV6 has been reported to be involved in solid tumors. ETV6 played an important role in the tumorigenesis of non-small cell lung cancer (NSCLC) and nasopharyngeal carcinoma (NPC) [18, 40], ETV6 overexpresseion in NSCLC tissues and NPC biopsies was correlated with increased patient age and poor prognosis, poor disease-free rates and poor overall survival rates of NSCLC and NPC [18, 40]. It has been suggested that ETV6 knockdown could inhibit migration and invasion abilities of breast cancer Hs578T cells [41]. However, the role of ETV6 in hepatocarcinoma remains unclear. Our current work showed that the upregulation of ETV6 potentially promoted the clinical development and progression of hepatocarcinoma patients and enhanced hepatocarcinoma cells aggressiveness (Fig. 1a, b). Furthermore, we first investigated the effects of ETV6 deregulation on the malignant behaviors of HCC cells. The overexpression of ETV6 remarkably promoted the in vitro migration and invasion capacities of HCCLM3 and HuH7 cells (Fig. 5c), while, its knockdown significantly inhibited the migration and invasion abilities of HCCLM3 and HuH7 cells (Fig. 5d). ETV6 affects cancer cells’ malignancy by interrupting cellular F-actin polymerization. ETV6 overexpression resulted in an obvious increase of the F-actin microfilament (Fig. 5e), while, ETV6 knockdown led to an apparent decrease of the F-actin microfilament (Fig. 5e). Our results demonstrated that ETV6 as a tumor promoter in the development and progression of hepatocarcinoma. The movement of cancer cells from one site to another location is a complex process requiring dramatic remodeling of cell cytoskeleton, microfilaments (actin) is major cytoskeletal filaments, tumor cell metastasis as a process of cell motility can be broken down into four steps: protrusion, adhesion, contraction and retraction, abnormal expression or regulation of cytoskeleton components could affect the adhesion with ECM, thus affecting migration and invasion [42–44]. Our results demonstrated that by enhancing the cytoskeleton F-actin polymerization, ETV6 upregulation enhanced the aggressiveness of hepatocarcinoma cells. ETV6 upregulation is a potential indicator for hepatocarcinoma malignancy.

Because of its role as a multi-functional adaptor protein in signal transduction, CRKL deregulation is involved in a variety of cancers. It is an attractive target for the diagnostics, treatment and prognosis of certain cancers [20]. Previously, we detected that endogenous expression level of CRKL in the tumorous tissues from 12 hepatocarcinoma patients was upregulated by 66.7% compared to the paired non-tumor live tissues [26]. In the current study, the protein level of CRKL was upregulated by 79.6% (*P* = 0.0041) in the tumor from 16 additional hepatocarcinoma patients (Fig. 2a). IHC results also showed CRKL highly expressed in hepatocarcinoma tissues in over 58.3% (28 out of 48) of the patient samples (Fig. 2c, Table 1). Consistently, CRKL was more abundant in hepatocarcinoma cell lines HepG2, HCCLM3 and HuH7 compared with normal liver LO2 cells (Fig. 2b). CRKL expression level affected the malignant behaviors of hepatocarcinoma cell lines. Its overexpression promoted the migration (Fig. 6b) and invasion (Fig. 6c) abilities of HepG2, HCCLM3 and HuH7 cells. Consistent with the effect of ETV6 on cytoskeleton, CRKL overexpression led to an increase in the intracellular microfilament of the F-actin cytoskeleton (Fig. 6d). Hence, our current work establishes a tumor promoter role for CRKL in hepatocarcinoma progression and hepatocarcinoma cells aggressiveness.

miR-429 is a member of the miR-200 family including miR-200a, miR-200b, miR-200c, miR-141 and miR-429 [45]. miR-429 abnormal expression is linked to osteosarcoma, renal cancer, ovarian cancer, glioma, breast cancer, oral squamous cell carcinoma, gastric cancer, esophagus cancer, cervical cancer, bladder cancer, lung cancer, prostate cancer, colon cancer [31]. It shows suppression or promotion effects on tumor development, invasion, metastasis, apoptosis and drug-resistance depending on the tumor type and subtype [46–49]. It is a potential indicator for the diagnosis, treatment and prognosis of certain tumors [31, 50–52]. Previously, we detected that the endogenous expression level of miR-429 in the tumorous tissues from 12 hepatocarcinoma patients was downregulated compared to the paired non-tumor live tissues [26]. Furthermore, the expression level of miR-429 was downregulated by 27.5% (*P* = 0.0136) in the tumor from 16 additional hepatocarcinoma patients (Fig. 3a) and in hepatocarcinoma HepG2, HCCLM3 and HuH7 cell lines (Fig. 3b). The deficiency of miR-429 promoted tumorigenesis and malignancy of hepatocarcinoma, and miR-429 played a tumor suppressive role in hepatocarcinoma progression and hepatocarcinoma cells’ aggressiveness. The above results were consistent with our previous work showing that miR-429 overexpression or knockdown significantly reduced or increased in vitro migration and invasion capacities of HepG2 cells, respectively [26]. Moreover, miR-429 overexpression resulted in an obvious decrease of F-actin microfilament in HepG2 cells [26]. Also, since the dysexpressions of both ETV6 and CRKL could also affect the malignant behaviors through F-actin polymerization, it is a logical step to connect miR-429 with ETV6 and CRKL in hepatocarcinoma progression.

ETV6 is known to forms fusion protein by chromosomal translocations with ABL [9]. CRKL is a substrate protein for ABL, and the Tel-ABL fusion protein can form complexes with CRKL in leukemia [10]. The HLH domain of ETV6 could mediate dimerization of ABL with subsequent activation of the ABL kinase domain leading to tyrosine-phosphorylation of CRK, and the Tel-Abl-CRKL complexes activated Ras, MAPK/ERK and AKT/PKB [10]. Consistently, our previous study found miR-429 suppressed migration and invasion by targeting CRKL via inhibiting the Raf/MEK/ERK pathway and EMT [26]. However, whether ETV6 associates with CRKL in solid tumors is unknown. Our work has for the first shown that ETV6 overexpression was positively correlated with CRKL upregulation in hepatocarcinoma tissues and cell lines (Fig. 4a). Moreover, the overexpression and knockdown of CRKL positively upregulated (Fig. 7a) and downregulated (Fig. 7b) ETV6 expression levels in HepG2, HCCLM3 and HuH7 cells. Concordantly, CRKL expression level in these cells could also be positively regulated by ETV6 dysexpression (Fig. 7c). In addition, we have shown that ETV6 directly binds to CRKL by Co-IP assay further indicated the direct interaction between ETV6 and CRKL (Fig. 7d).

The *ETV6-NTRK3* (neurotrophic tyrosine kinase receptor type 3) fusion gene was common in cancers such as secretory breast carcinoma, mesoblastic nephroma and congenital fibrosarcoma [53–55], ETV6-NTRK3 fusion oncoprotein could initiate breast cancer from committed mammary progenitors via activation of Wnt (wingless/integrated) signaling pathway [53], committed mammary progenitors, rather than mammary stem cells, are direct targets of transformation, gene fusions with kinase activities has revolutionized targeted therapies for cancer, modeling chromosomal rearrangements in human epithelial tumors could provide novel insights into their pathogenesis, preclinical testing and therapy. The abnormal regulation of Wnt signaling pathway is a major and early carcinogenic event which can be triggered by CTNNB1 (cadherin-associated protein, beta 1), accumulating evidences have demonstrated that activation of Wnt signaling can act in concert with other oncogenes, TGF-β (transforming growth factor-β), HGF/c-Met (hepatocyte growth factor/CENP-meta) pathway, HIF-1α (hypoxia inducing factor 1)/EMT pathway and IGF-1 (insulin-like growth factor 1) pathway, crosstalk between the HGF/c-Met pathway and the Wnt pathway might also contribute to the progression of HCC, CTNNB1 enhanced c-Met-stimulated FAK (focal adhesion kinase) activation and synergistically induced the activation of the AKT/ERK pathway [56]. ETV6-CRKL complexes could also activate AKT/ERK pathway. These results demonstrated ETV6 affected tumor progression via AKT/ERK pathway.

The current work also establishes the association of ETV6 with miR-429 in hepatocarcinoma. First, we found that ETV6 upregulation was negatively correlated with miR-429 deficiency in tumorous tissues from hepatocarcinoma patients (Fig. 4c). Second, in comparison with normal liver LO2 cells, a deficiency of miR-429 was measured in hepatocarcinoma cell lines HepG2, HCCLM3 and HuH7 (Fig. 4c). Third, ETV6 negatively regulated miR-429 expression levels in HepG2, HCCLM3 and HuH7 cells (Fig. 8a). ETV6 also acts as a strong transcriptional repressor by directly binding to target genes through an ETS DNA-binding domain to repress its transcription [11, 12]. The ETS domain is responsible for DNA binding to the ETS-binding consensus site (EBS) that contains a purine-rich GGAA/T core motif [50]. Bioinformatics combined with the ChIP assay revealed that by directly binding to the DNA promoter region GGAGGAAGCA at the 696–705 bp site of miR-429, ETV6 reversely mediated the expression of miR-429 in hepatocarcinoma cells (Fig. 8b).

Previously, we reported that miR-429 negatively regulates CRKL expression in HepG2 cells by selectively targeting its 3′-UTR at the 3728–3735 bp site and we established the primary association of miR-429 with CRKL in hepatocarcinoma [26]. Herein, we demonstrated that the upregulation of CRKL negatively correlated with miR-429 deficiency potentially promoted the development and progression of hepatocarcinoma patients and the aggressiveness of hepatocarcinoma cells (Fig. 4b). Meanwhile, we further demonstrated that CRKL negatively regulated miR-429 expression in hepatocarcinoma cell lines. CRKL overexpression and knockdown could decrease (Fig. 9a) and increase (Fig. 9b) the expression levels of miR-429 in HepG2, HCCLM3 and HuH7 cells.

**Fig. 10:**
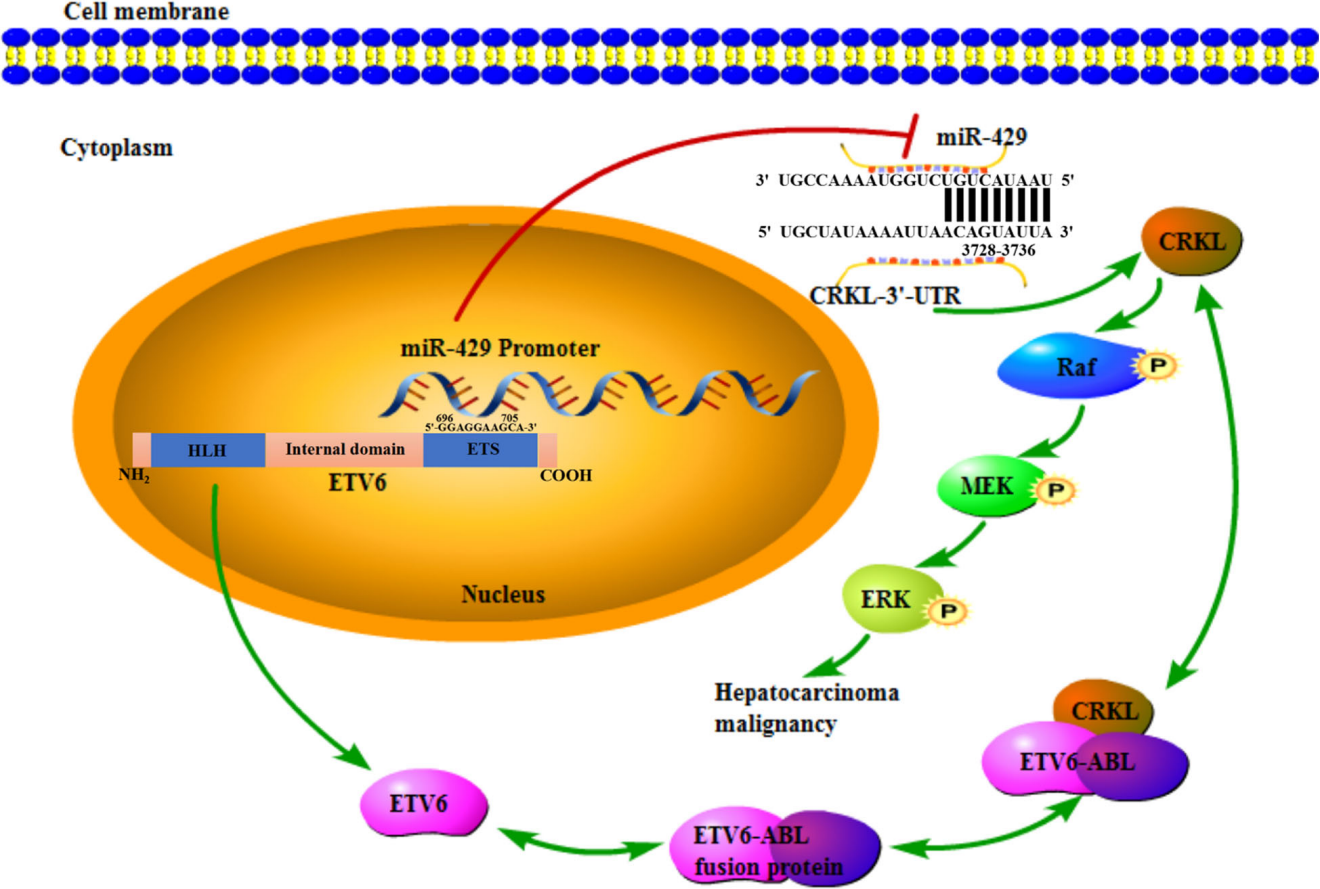
The schematic regulation mechanism of the ETV6-miR-429-CRKL regulatory circuitry in HCC. ETV6 directly binds to the promoter region of miR-429 to positively regulate the expression of CRKL by selectively targeting *CRKL*-3′-UTR; ETV6 upregulates CRKL expression by directly binding to CRKL; which in turn CRKL upregulation promotes ETV6 expression; CRKL overexpression potentially promotes hepatocarcinoma malignancy via activating the ERK pathway

As illustrated in Fig. 10, The ETS domain of ETV6 directly binds to the DNA promoter region GGAGGAAGCA at the 696–705 bp site of miR-429 to suppress its expression, then miR-429 downregulation promotes CRKL expression by selectively targeting *CRKL*-3′-UTR at the 3728–3735 bp site. Meanwhile, ETV6 forms complexes with CRKL and promotes CRKL expression, which in turn promotes ETV6 expression. Furthermore, CRKL overexpression potentially promotes hepatocarcinoma malignancy by activating ERK pathway.

Hepatocarcinogenesis is a very complex biological process associated with environmental risk factors and hereditary factors, including abnormal activation of Wnt/β-catenin, MAPK, AKT, and ERK signaling pathways, and the balance between activation and inactivation of proto-oncogenes and anti-oncogenes, and the differentiation of liver cancer stem cells [57]. Ras/Raf/ERK is the most crucial pathway in the development and progression of HCC. Signals from membrane-binding tyrosine kinase receptors, such as endothelial growth factor receptor (EGFR), insulin-like growth factor receptor (IGFR), c-Met and PDGFR, are transduced to cell nucleus through Ras/Raf/ERK pathway to regulate multiple cellular functions [57]. Molecule-targeted therapy, a new approach for the treatment of HCC, blocks tumor cells growth by interfering with the molecules required for carcinogenesis. Effective drugs blocking Ras/Raf/ERK signaling are still at exploratory stage, except for sorafenib that has activity inhibiting Raf [57]. Sorafenib is an oral multi-targeted tyrosine kinase inhibitor (TKI) targeting PDGFR, vascular endothelial growth factor (VEGFR), fibroblast growth factor receptor (FGFR), Raf, which is a gold quality standard in the first line treatment of advanced HCC patients. Cancer biomarkers have been widely used for prediction of prognosis and treatment response in certain tumors [58, 59]. Predictive biomarkers can be best explored in the setting of properly designed clinical trials, and it is important to develop biomarkers to narrow down the subgroup of the patients who have a survival benefit from sorafenib treatment.

Our results showed that an ETV6-miR-429-CRKL regulatory circuitry contributes to aggressiveness of HCC by activating Ras/Raf/ERK pathway. ETV6 is known to forms fusion protein with PDGFR, CRKL can function in cellular signaling cascades by directly forms complex with downstream receptor protein to regulate cellular tyrosine kinase activity, meanwhile, some miRNAs have been evaluated for the prediction of response to sorafenib treatment in HCC [60]. While, sorafenib as a TKI could directly inhibit PDGFR and Raf, we speculate ETV6, CRKL, miR-429 might as novel biomarkers for the predication of sorafenib response in HCC, in the future study, we will clarified and validated whether ETV6, CRKL, miR-429 could predict the response of HCC to sorafenib treatment, and we expect ETV6, CRKL, miR-429 could help identify the subclass of HCC patients who would likely respond to sorafenib treatment. Molecule-targeted therapy will gradually become a new favorite for treatment of HCC, and also represent the future developmental direction of treatment of HCC. Furthermore, fundamental research breakthroughs will create more effective methods for HCC targeted therapy.

## Conclusions

CRKL, ETV6 acts as tumor promotors and miR-429 acts as a tumor suppressor in HCC, their dysexpressions are involved in the carcinogenesis and progression of hepatocarcinoma. We have uncovered a novel ETV6-miR-429-CRKL regulatory circuitry that contributes to hepatocarcinoma tumorigenesis. The newly identified ETV6-miR-429-CRKL regulatory circuitry from the current work provides new insights into the underlying mechanism of pathogenesis in HCC and represents a potential therapeutic target for diagnosis and treatment of HCC.

## Supplementary information

**Additional file 1.**

## Data Availability

The data supporting the conclusions of this article are included within the article.

## Abbreviations

HCC: Human hepatocellular carcinoma
CRKL: V-crk sarcoma virus oncogene homologue (avian)-like
ECM: Extracellular matrix
HLH: Helix-loop-helix
PNT: Pointed domain
SAM: Sterile alpha motif domain
ETS: E-Twenty Six
ETV6: ETS variant gene 6
Tel: Translocation ets leukaemia
EBS: ETS-binding consensus site
PDGFR: Platelet-derived growth factor receptor
ABL: V-abl Abelson murine leukemia viral oncogene
EVI-1: Ecotropic virus integration site 1
RUXN1: Runt-related transcription factor 1 gene
Ras: Rat sarcoma
MAPK/ERK: Mitogen-activated protein kinase/extracellular regulated protein kinases
PKB/AKT: Protein kinase B/Serine/threonine kinase
BCAR1: Breast cancer anti-estrogen resistance 1
GAB: Grb2-associated binder protein
Pax: Paired boxed gene
GEF: Guanine nucleotide exchange factor
C3G: 3-domain-binding guanine nucleotide exchange factor
BCR: B-cell receptor
SOS: Son of sevenless
Raf: Rapidly accelerated fibrosarcoma
MEK: Mitogen-activated extracellular signal-regulated kinase
miRNAs: MicroRNAs
3′-UTR: 3′-untranslated region
EMT: Epithelial-mesenchymal transition
HBV: Hepatitis B virus
AFP: Alpha fetoprotein
DMEM: Dulbecco′s modified Eagle′s medium
FBS: Fetal bovine serum
WB: Western blotting
RT: Room temperature
NC: Nitrocellulose
ACTB: β-actin
GAPDH: Glyceraldehyde-3-phosphate dehydrogenase
HRP: Horseradish peroxidase
PBS: Phosphate buffered solution
IHC: Immunohistochemistry
qRT-PCR: Quantitative real-time PCR
TRITC: Tetramethyl rhodamin isothiocyanate
RIPA: Radio-immunoprecipitation assay
SDS-PAGE: Sodium dodecyl sulfate-polyacrylamide gel electrophoresis
TBST: Tris-buffered-saline-tween 20
PMSF: Phenylmethanesulfonyl fluoride
BSA: Bovine serum albumin
ECL: Electrochemiluminescence
DAB: Diaminobenzidin
siRNA: Small interfering RNA
Co-IP: Co-immunoprecipitation
ChIP: Chromatin immunoprecipitation
NSCLC: Non-small cell lung cancer
NPC: Nasopharyngeal carcinoma
SH2: Src homology2
SH3N: N-terminal SH3
SH3C: C-terminal SH3
ccRCC: Clear cell renal cell carcinoma
BM: Bone marrow
CML: Chronic myeloid leukemia
PB: Peripheral blood
NTRK3: Neurotrophic tyrosine kinase receptor type 3
Wnt: Wingless/Integrated
CTNNB1: Cadherin-associated protein, beta 1
TGF-β: Ttransforming growth factor-β
HGF/c-Met: Hepatocyte growth factor/CENP-meta
HIF-1α: Hypoxia inducing factor 1
IGF-1: Insulin-like growth factor 1
FAK: Focal adhesion kinase
EGFR: Growth factor receptor
IGFR: Insulin-like growth factor receptor
TKI: Tyrosine kinase inhibitor
VEGFR: Vascular endothelial growth factor
FGFR: Fbroblast growth factor receptor
