# Correction to: A novel ETV6-miR-429-CRKL regulatory circuitry contributes to aggressiveness of hepatocellular carcinoma

**DOI:** 10.1186/s13046-021-01860-7

**Published:** 2021-02-17

**Authors:** Chunmei Guo, Chao Gao, Dongting Zhao, Jiahui Li, Jinxia Wang, Xujuan Sun, Qinlong Liu, Lihong Hao, Frederick T. Greenaway, Yuxiang Tian, Shuqing Liu, Ming-Zhong Sun

**Affiliations:** 1 Department of Biotechnology, College of Basic Medical Sciences, Dalian Medical University, Dalian, 116044 China; 2 Department of Biochemistry, College of Basic Medical Sciences, Dalian Medical University, Dalian, 116044 China; 3 Department of General Surgery, The Second Affiliated Hospital, Dalian Medical University, Dalian, 116044 China; 4 Department of Histology and Embryology, College of Basic Medical Sciences, Dalian Medical University, Dalian, 116044 China; 5 Carlson School of Chemistry and Biochemistry, Clark University, Worcester, MA 01610 USA

**Correction to: J Exp Clin Cancer Res 39, 70 (2020)**

**https://doi.org/10.1186/s13046-020-01559-1**

Following publication of the original article [1], the authors identified a minor error in image-typesetting in Fig. 5; specifically in Fig. 5d, the incorrect representative image of invasion of HuH7-siNC was used. The corrected figure is given below. The authors state that the data processing and statistical analysis was based on the correct invasion image; therefore the correction has no effect to the final conclusion.

**Incorrect Fig. 5:**

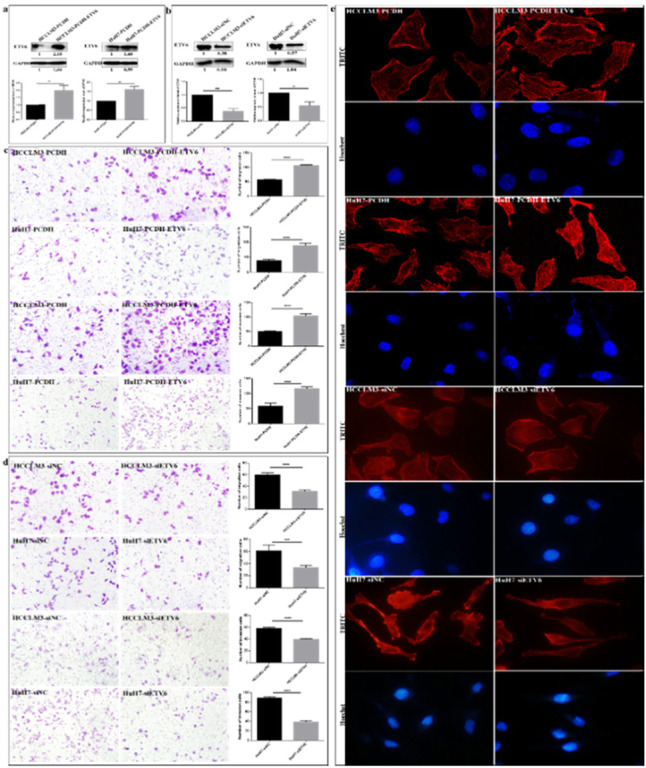



**Correct Fig. 5:**

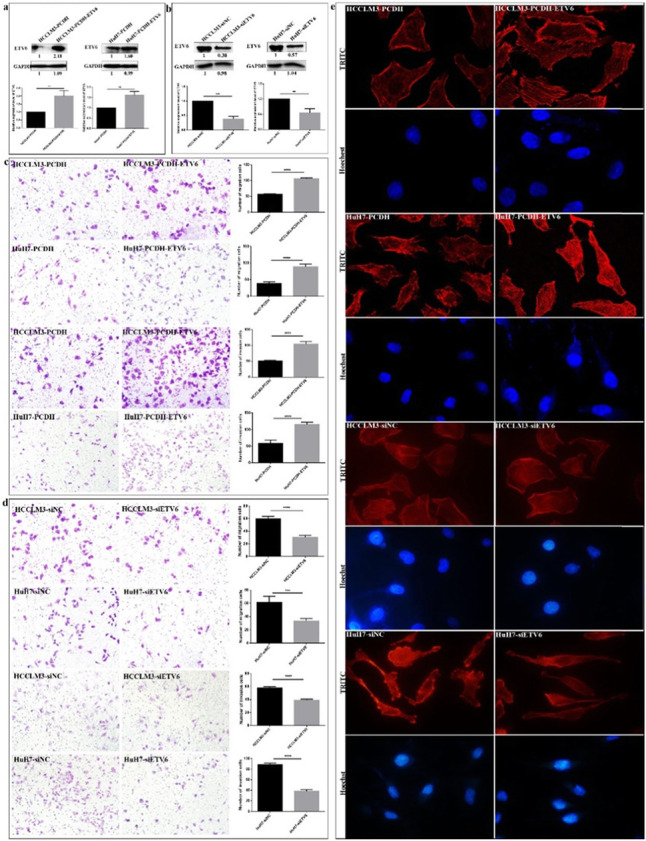



The authors regret and apologize for any inconvenience to readers arising from the error. The original article [1] has been corrected.

## References

[1] Guo C, Gao C, Zhao D (2020). A novel ETV6-miR-429-CRKL regulatory circuitry contributes to aggressiveness of hepatocellular carcinoma. J Exp Clin Cancer Res.

