# Supplementary material for: A novel ETV6-miR-429-CRKL regulatory circuitry contributes to aggressiveness of hepatocellular carcinoma
Source: J Exp Clin Cancer Res. 2020 Apr 23;39:70. doi: 10.1186/s13046-020-01559-1 (PMC7178969; doi:10.1186/s13046-020-01559-1)
Supplement: Supplementary file 1 — Additional file 1. [file 13046_2020_1559_MOESM1_ESM.docx]

**The corresponding whole western blot images with all bands for each figures were provided as below:**

**1. Original western blotting images of Fig. 1a and Fig. 2a: We detected the protein expression level of ETV6 and CRKL in 42 pairs of matched liver cancer, bile duct cancer, pancreatic cancer, gallbladder adenocarcinoma and corresponding nontumor tissues, however, liver cancer tissues only including T1-T16. The uncropped images of individual patients were shown in Panel 1 to Panel 17, respectively.**


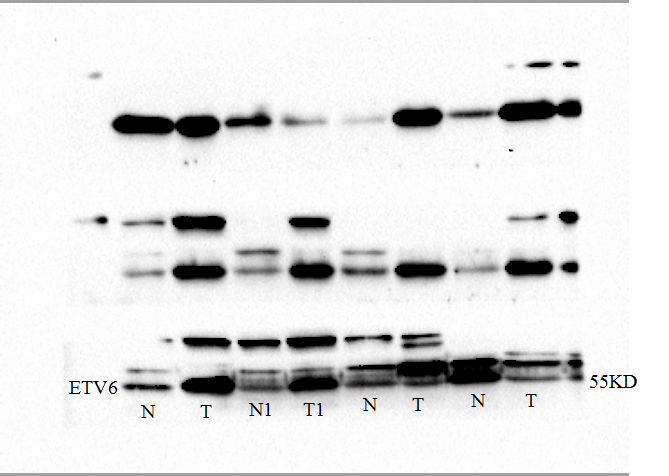


**Panel 1. Blotting images of ETV6 for samples from 1 patient (1)**


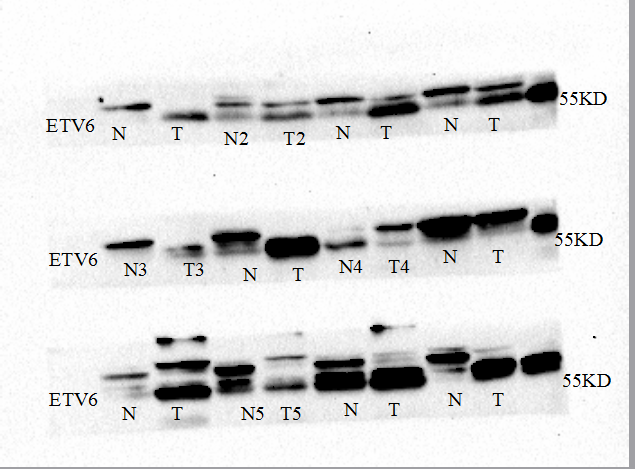


**Panel 2. Blotting images of ETV6 for samples from 4 patients (2-5)**


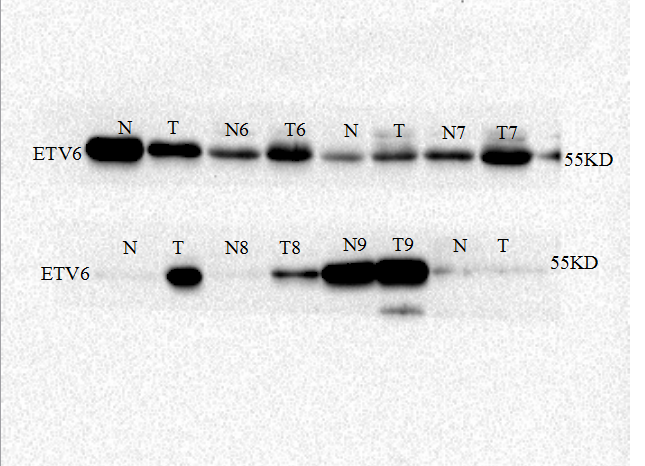


**Panel 3. Blotting images of ETV6 for samples from 4 patients (6-9)**


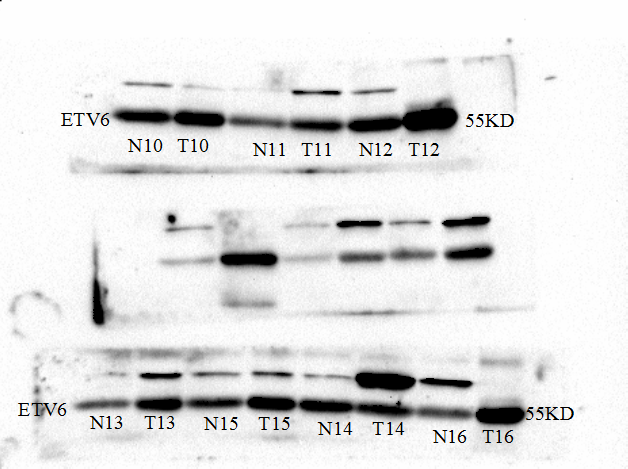


**Panel 4. Blotting images of ETV6 for samples from 7 patients (10-16)**


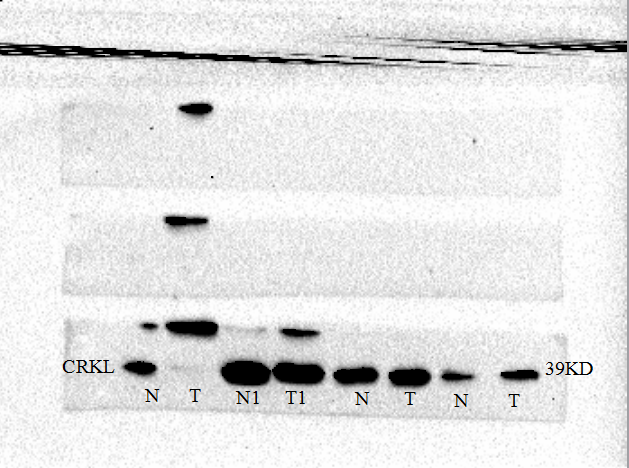


**Panel 5. Blotting images of CRKL for samples from 1 patient (1)**


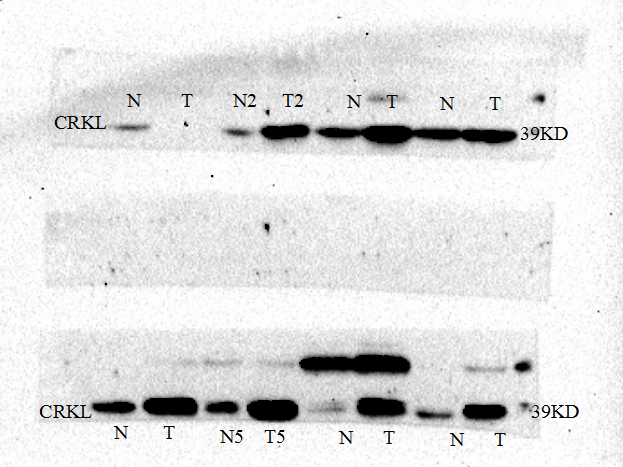


**Panel 6. Blotting images of CRKL for samples from 2 patients (2, 5)**


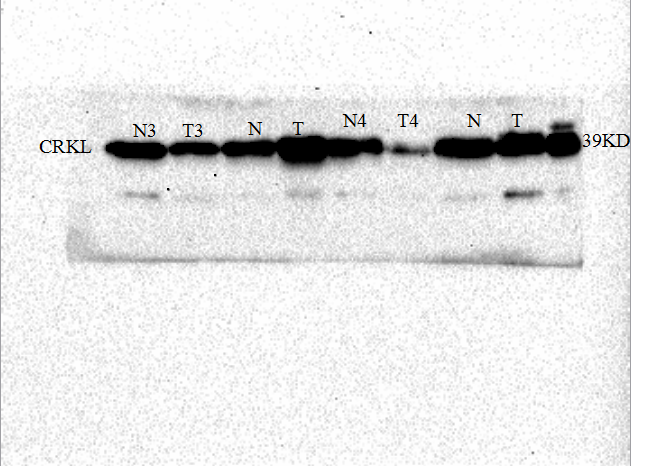


**Panel 7. Blotting images of CRKL for samples from 2 patients (3-4)**


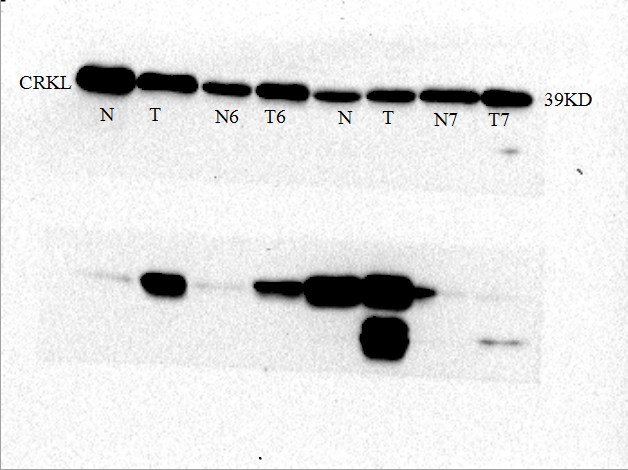


**Panel 8. Blotting images of CRKL for samples from 2 patients (6, 7)**


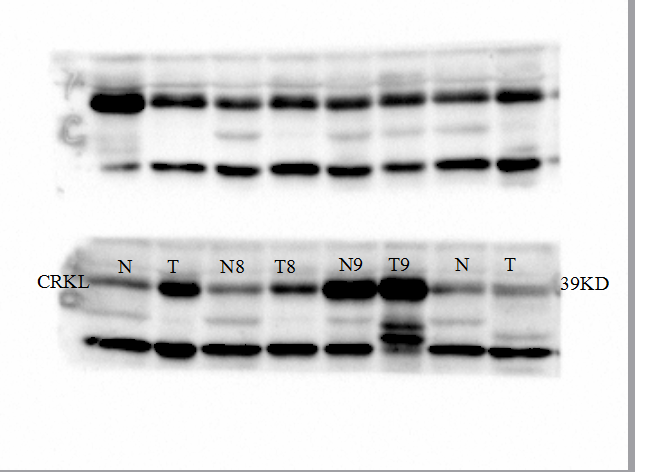


**Panel 9. Blotting images of CRKL for samples from 2 patients (8, 9)**


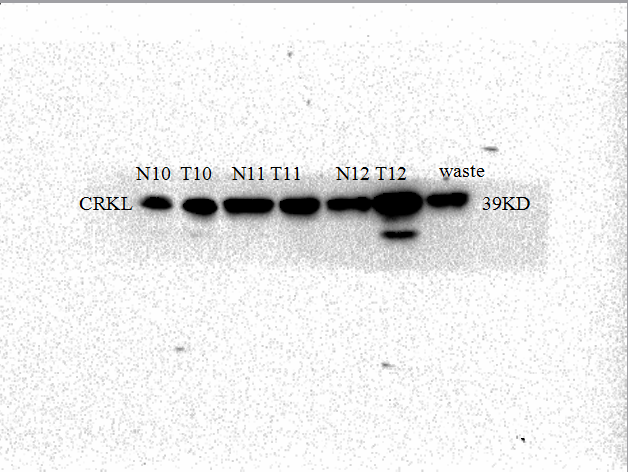


**Panel 10. Blotting images of CRKL for samples from 3 patients (10-12)**


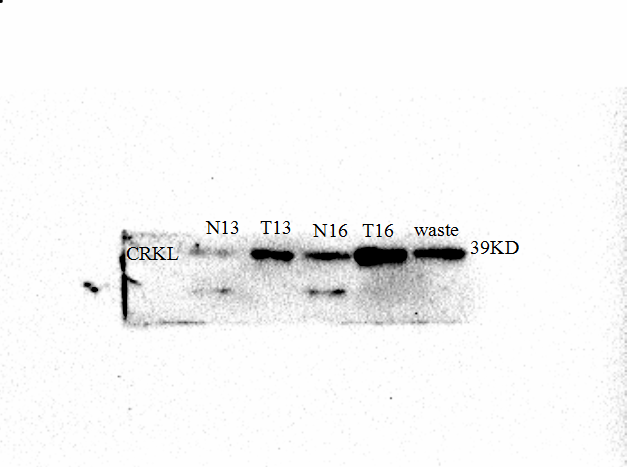


**Panel 11. Blotting images of CRKL for samples from 2 patients (13, 16)**


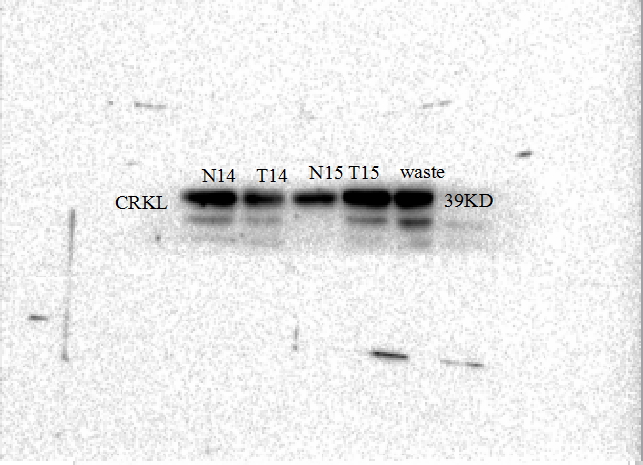


**Panel 12. Blotting images of CRKL for samples from 2 patients (14, 15)**


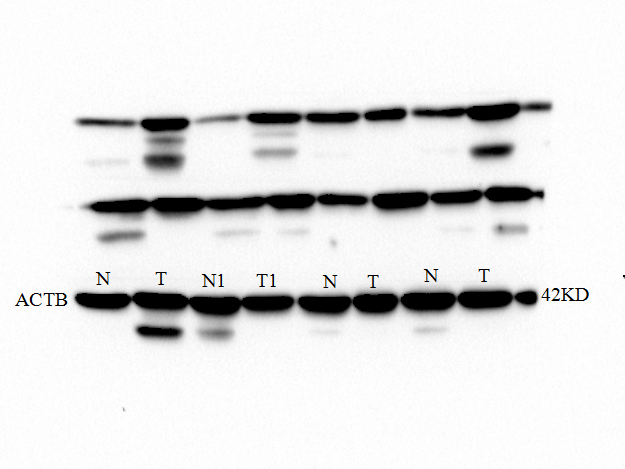


**Panel 13. Blotting images of ACTB for samples from 1 patient (1)**


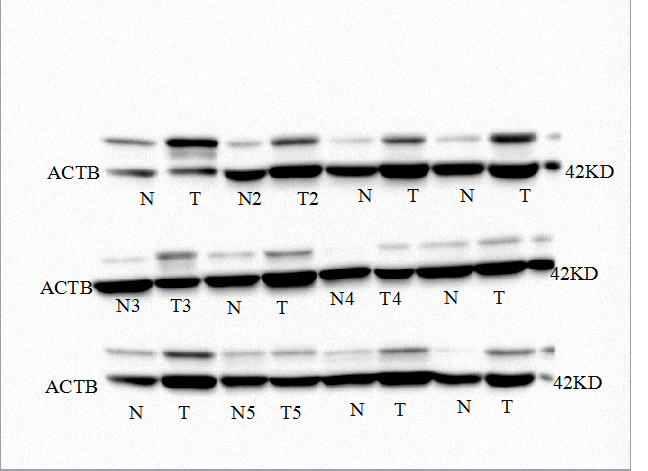


**Panel 14. Blotting images of ACTB for samples from 4 patient (2-5)**


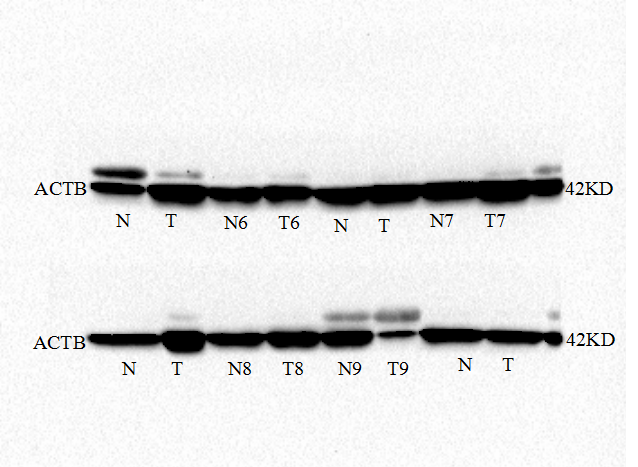


**Panel 15. Blotting images of ACTB for samples from 4 patient (6-9)**


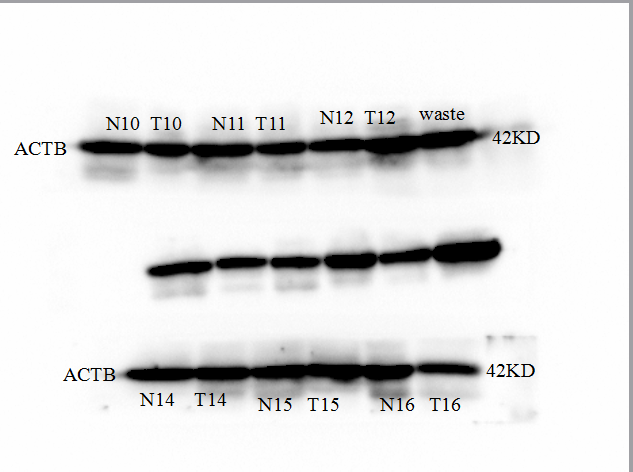


**Panel 16. Blotting images of ACTB for samples from 6 patient (10-12,14-16)**


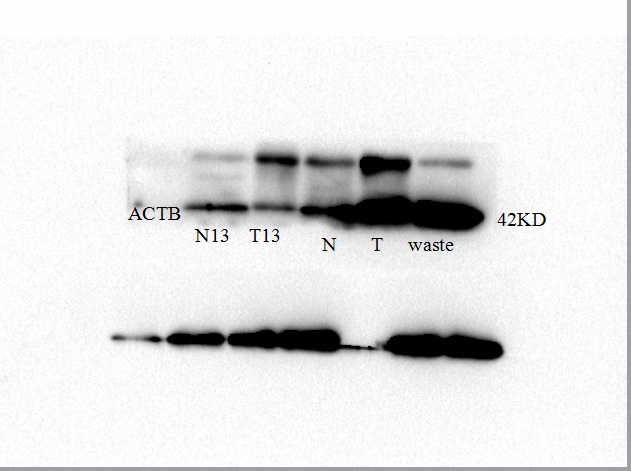


**Panel 17. Blotting images of ACTB for samples from 1 patient (13)**

**2. Original western blotting images of Fig. 1b and Fig. 2b: ETV6, CRKL and GAPDH expression levels in LO2, HepG2, HCCLM3 and HuH7 cells. The uncropped images of individual patients were shown in Panel 18 to Panel 20, respectively.**


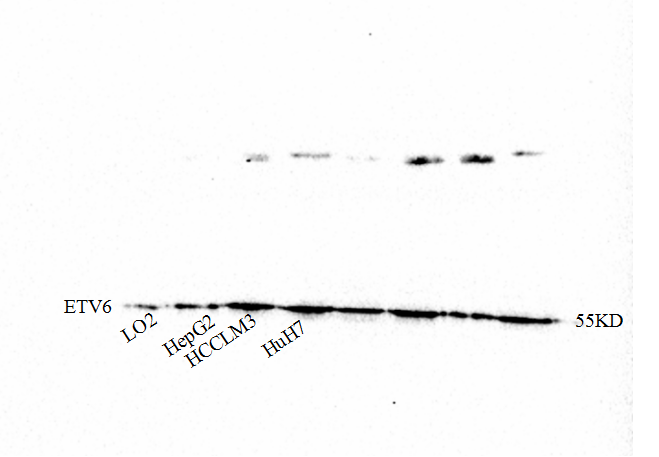


**Panel 18. Blotting images of ETV6**


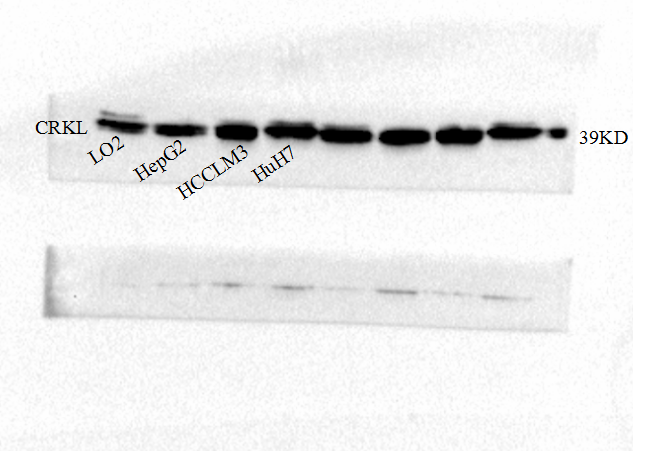


**Panel 19. Blotting images of CRKL**


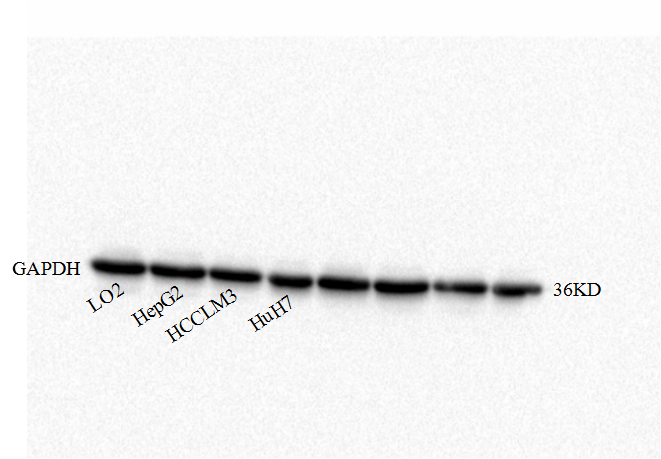


**Panel 20. Blotting images of GAPDH**

**3. Original western blot images for Fig. 5a and Fig. 5b: The uncropped western blotting images of ETV6 overexpression in HCCLM3 and HuH7 cells were shown in Panel 21 to Panel 22, respectively. The uncropped WB images of ETV6 knockdown in HCCLM3 and HuH7 cells were shown in Panel 23 to Panel 24, respectively.**


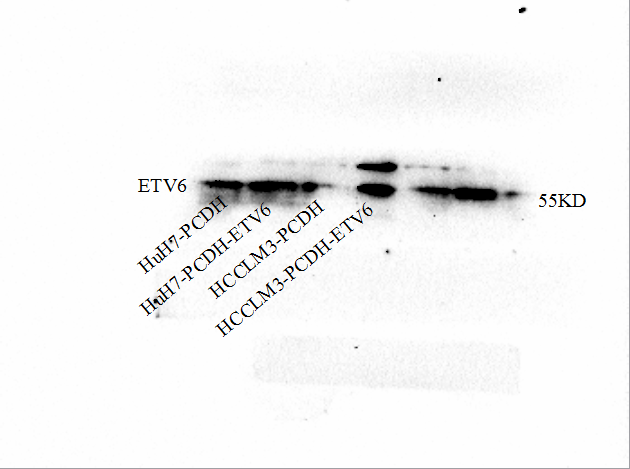


**Panel 21. Blotting images of ETV6 for ETV6 overexpression**


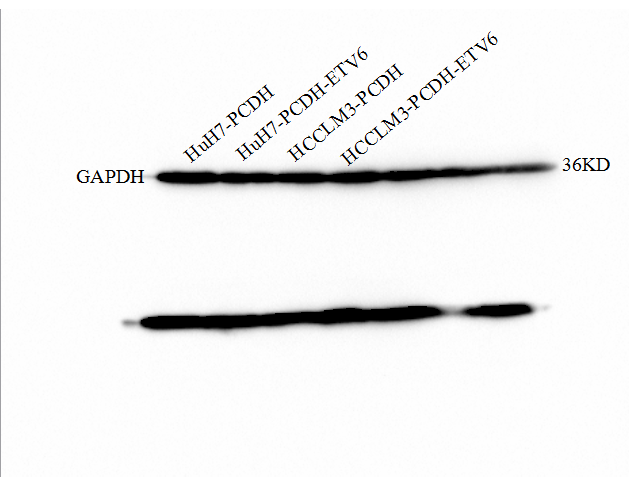


**Panel 22. Blotting images of GAPDH for ETV6 overexpression**


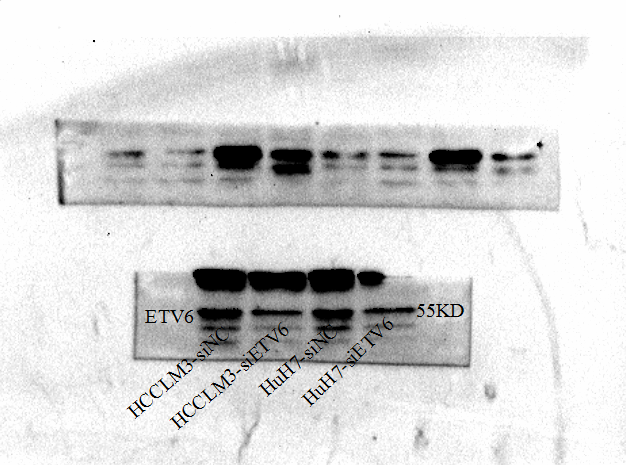


**Panel 23. Blotting images of ETV6 for ETV6 knockdown**


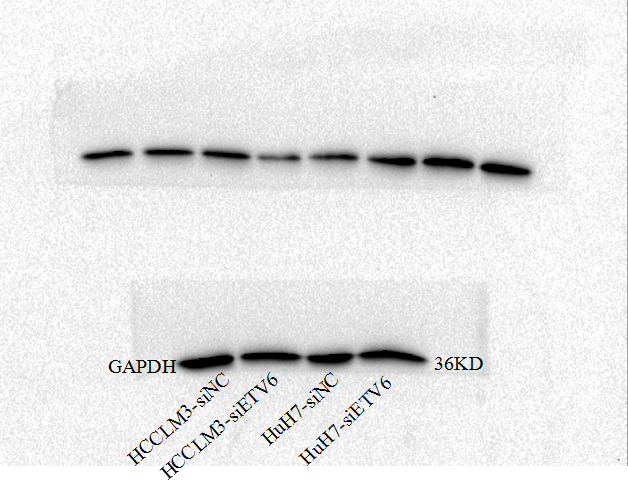


**Panel 24. Blotting images of GAPDH for ETV6 knockdown**

**4. Original western blot images for Fig. 6a: The uncropped western blotting images of CRKL overexpression in HepG2, HCCLM3 and HuH7 cells were shown in Panel 25 to Panel 28 , respectively.**


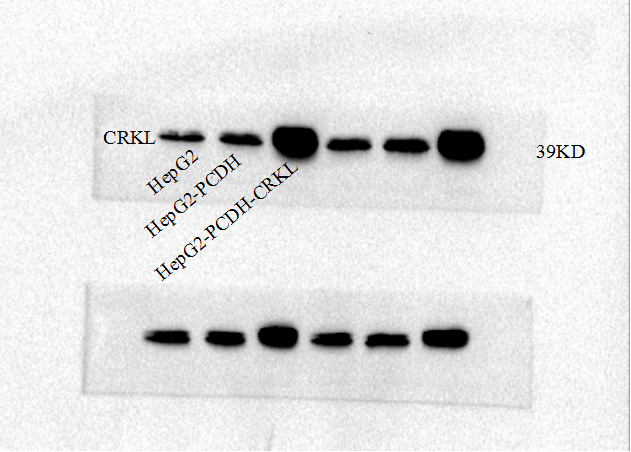


**Panel 25. Blotting images of CRKL for CRKL overexpression in HepG2 cell**


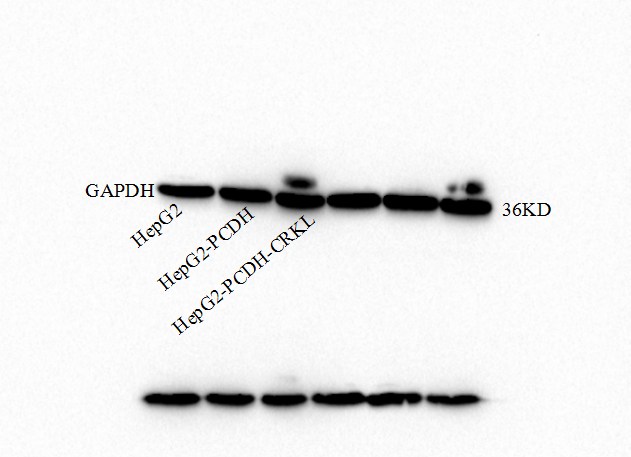


**Panel 26. Blotting images of GAPDH for CRKL overexpression in HepG2 cell**


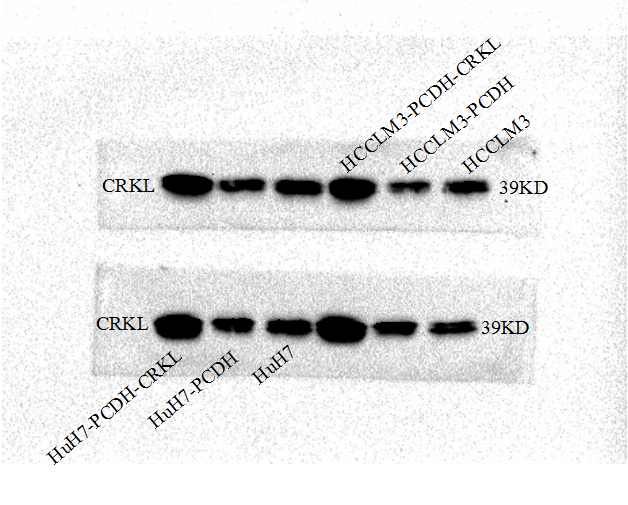


**Panel 27. Blotting images of CRKL for CRKL overexpression in HCCLM3 and HuH7 cells**


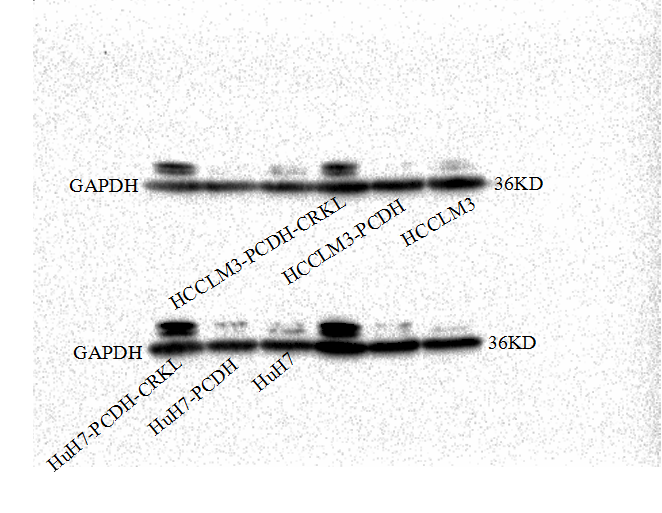


**Panel 28. Blotting images of GAPDH for CRKL overexpression in HCCLM3 and HuH7 cells**

**5. Original Western blot images for Fig. 7a, Fig. 7b, Fig. 7c and Fig. 7d: The uncropped western blotting images of CRKL overexpression in HepG2, HCCLM3 and HuH7 cells were shown in Panel 29 to Panel 34 for Fig 7a, respectively. The uncropped western blotting images of CRKL knockdown in HepG2, HCCLM3 and HuH7 cells were shown in Panel 35 to Panel 40 for Fig 7b, respectively. The uncropped western blotting images of ETV6 knockdown in HepG2, HCCLM3 and HuH7 cells were shown in Panel 41 to Panel 46 for Fig 7c, respectively. The uncropped Co-IP western blotting images of CRKL and ETV6 in HCCLM3 cells were shown in Panel 47 to Panel 53 for Fig 7d, respectively.**


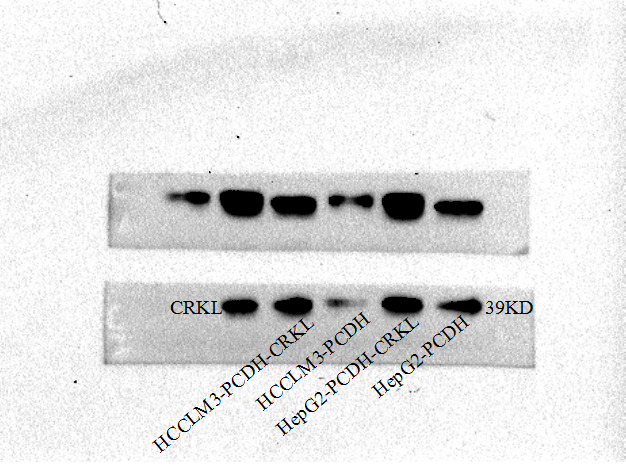


**Panel 29. Blotting images of CRKL for CRKL overexpression in HCCLM3 and HepG2 cells**

**
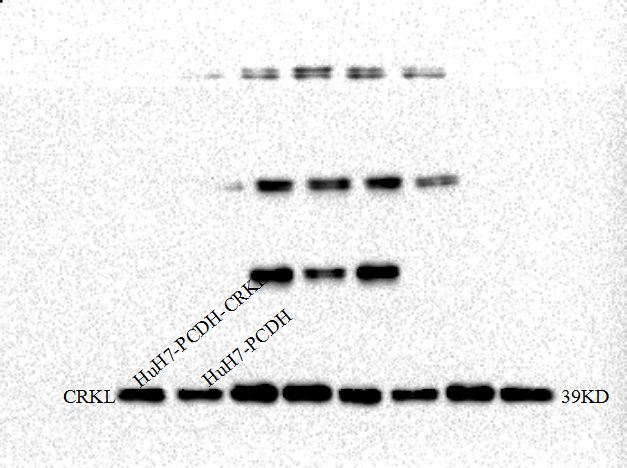
**

**Panel 30. Blotting images of CRKL for CRKL overexpression in HuH7 cell**


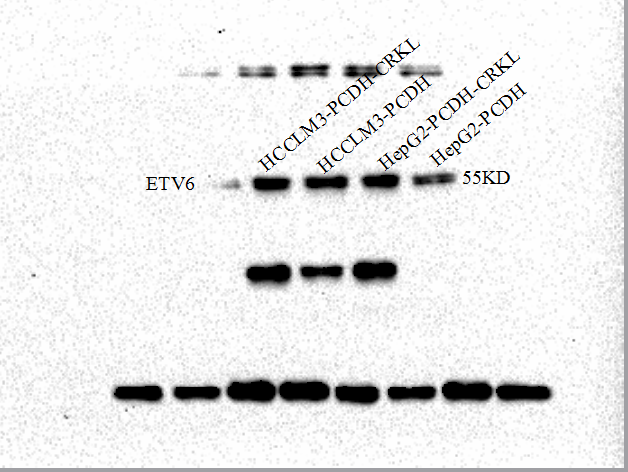


**Panel 31. Blotting images of ETV6 for CRKL overexpression in HCCLM3 and HepG2 cells**

**
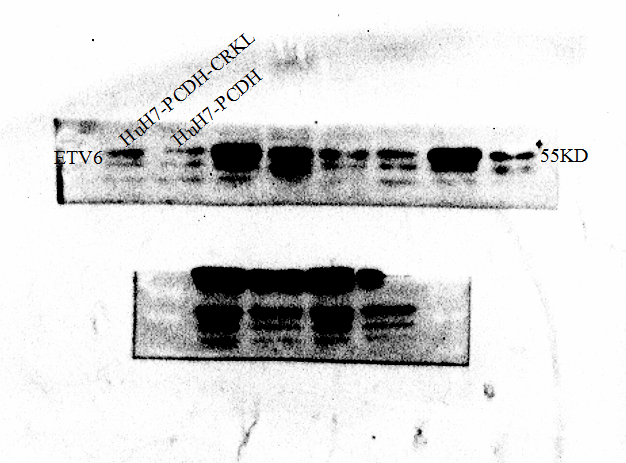
**

**Panel 32. Blotting images of ETV6 for CRKL overexpression in HuH7 cell**


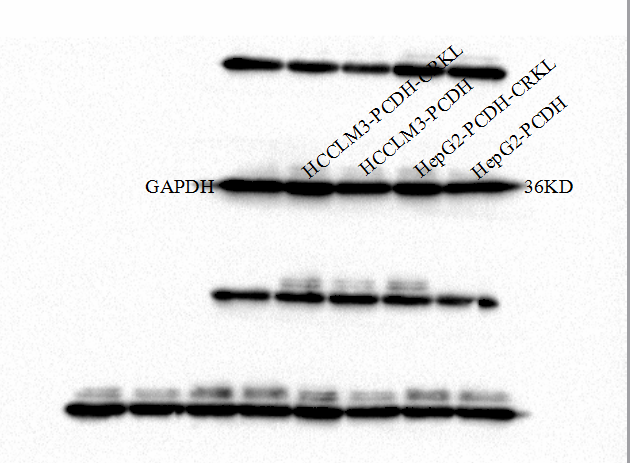


**Panel 33. Blotting images of GAPDH for CRKL overexpression in HCCLM3 and HepG2 cells**


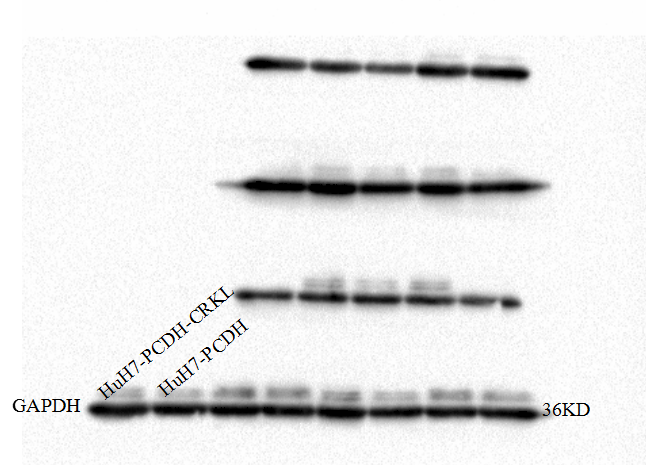


**Panel 34. Blotting images of GAPDH for CRKL overexpression in HuH7 cell**


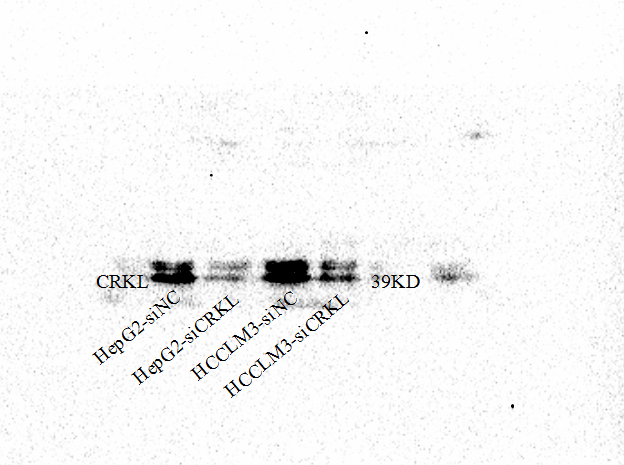


**Panel 35. Blotting images of CRKL for CRKL knockdown in HCCLM3 and HepG2 cells**


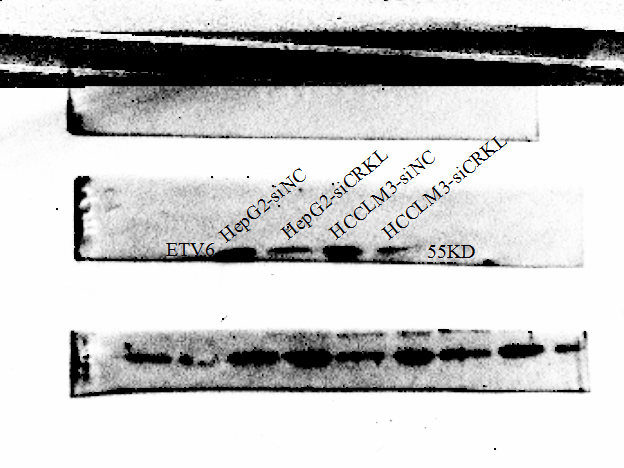


**Panel 36. Blotting images of ETV6 for CRKL knockdown in HCCLM3 and HepG2 cells**


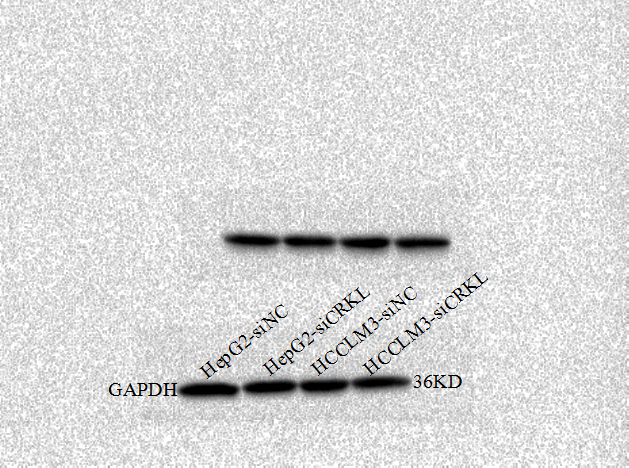


**Panel 37. Blotting images of GAPDH for CRKL knockdown in HCCLM3 and HepG2 cells**


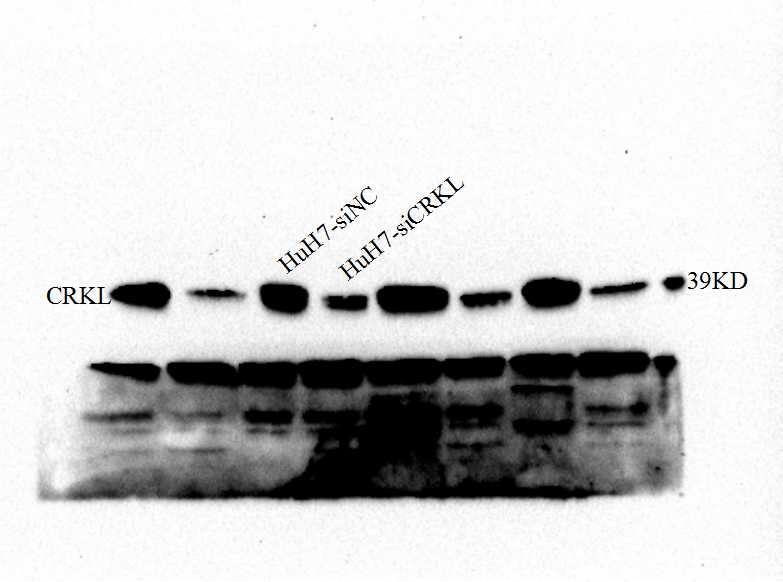


**Panel 38. Blotting images of CRKL for CRKL knockdown in HuH7 cell**


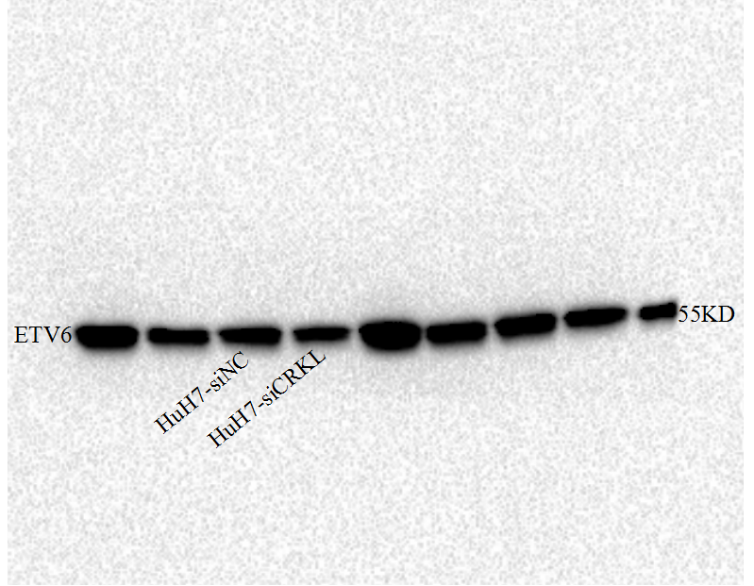


**Panel 39. Blotting images of ETV6 for CRKL knockdown in HuH7 cell**


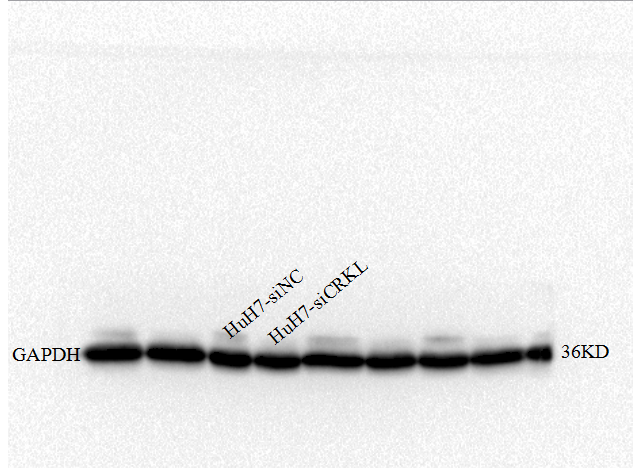


**Panel 40. Blotting images of GAPDH for CRKL knockdown in HuH7 cell**


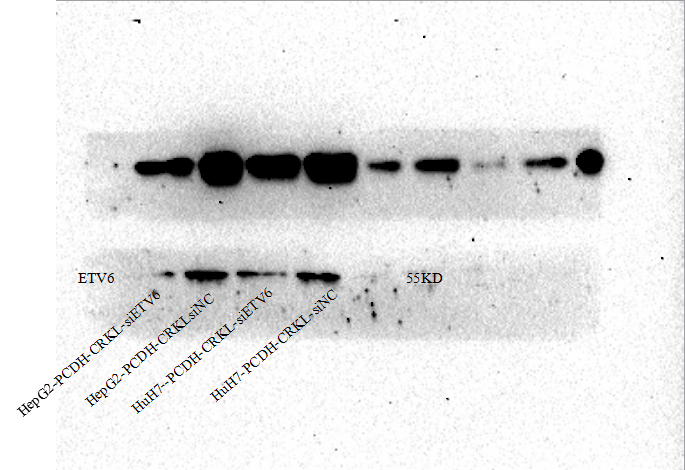


**Panel 41. Blotting images of ETV6 for ETV6 knockdown in HuH7 and HepG2 cells**


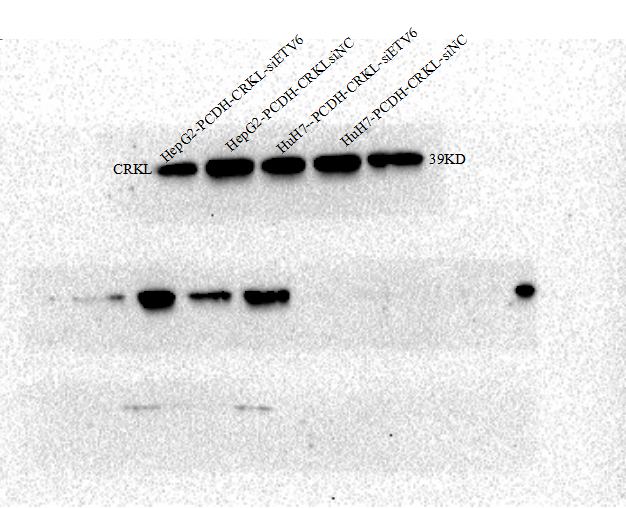


**Panel 42. Blotting images of CRKL for ETV6 knockdown in HuH7 and HepG2 cells**


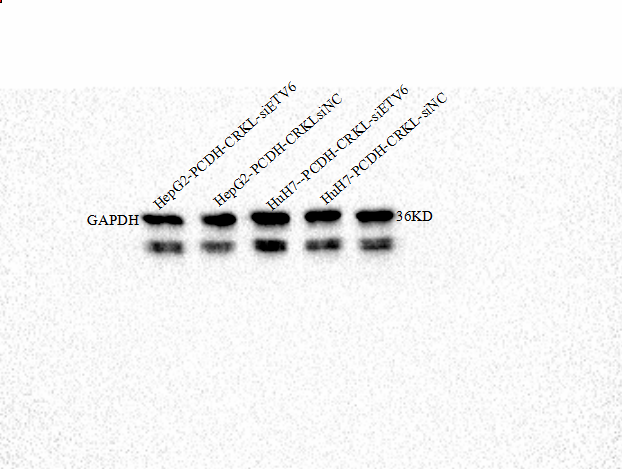


**Panel 43. Blotting images of GAPDH for ETV6 knockdown in HuH7 and HepG2 cells**


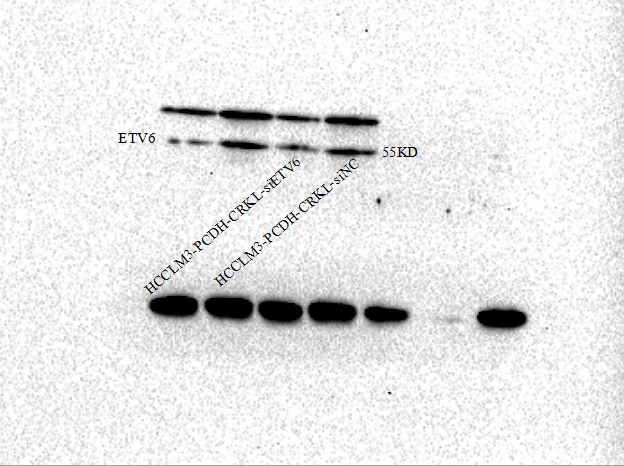


**Panel 44. Blotting images of ETV6 for ETV6 knockdown in HCCLM3 cell**


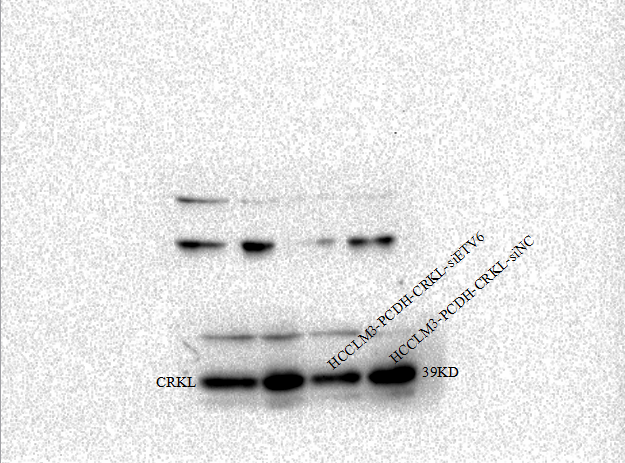


**Panel 45. Blotting images of CRKL for ETV6 knockdown in HCCLM3 cell**


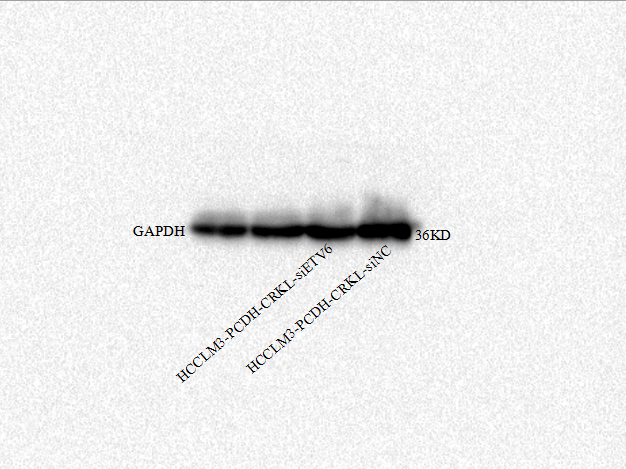


**Panel 46. Blotting images of GAPDH for ETV6 knockdown in HCCLM3 cell**


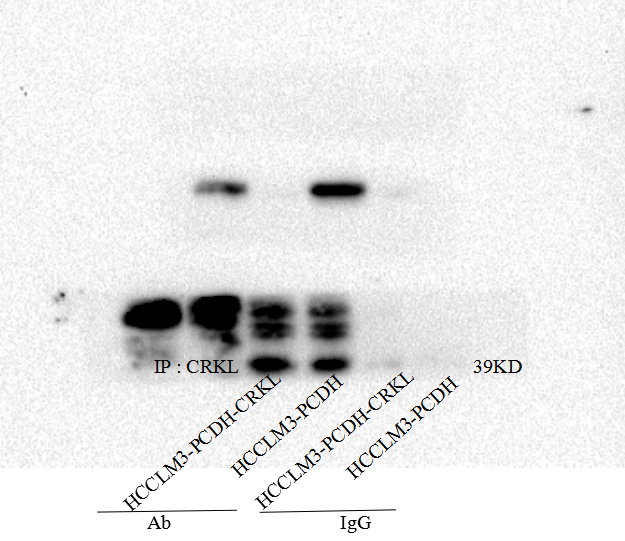


**Panel 47. Blotting images of IP CRKL for Ab and IgG**


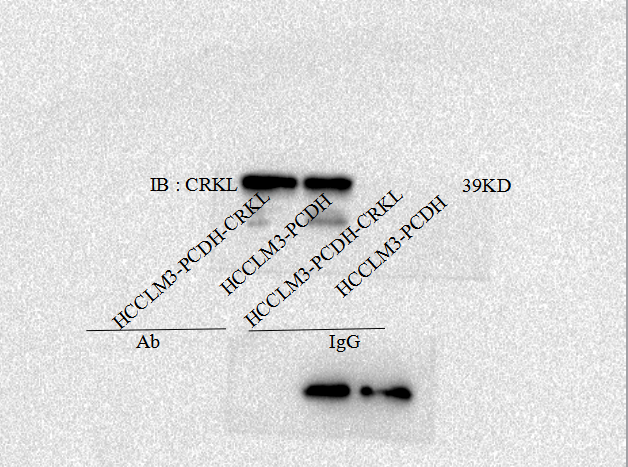


**Panel 48. Blotting images of IB CRKL for Ab and IgG**


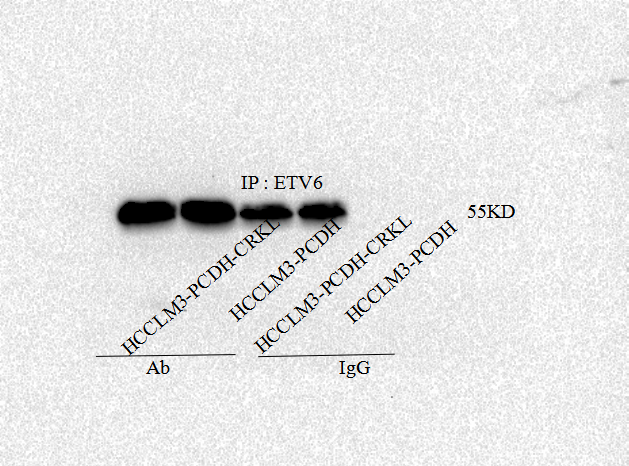


**Panel 49. Blotting images of IP ETV6 for Ab and IgG**


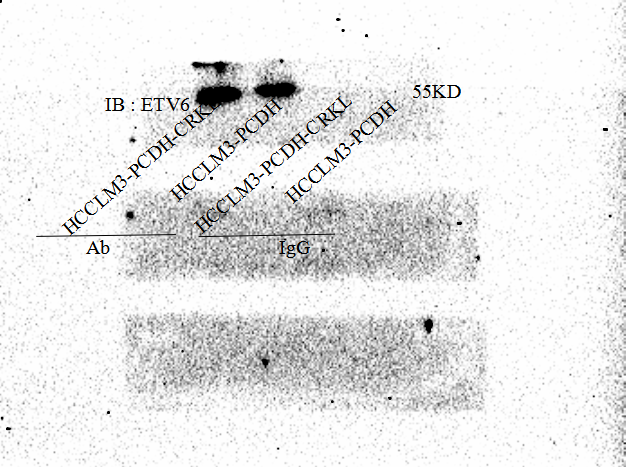


**Panel 50. Blotting images of IB ETV6 for Ab and IgG**


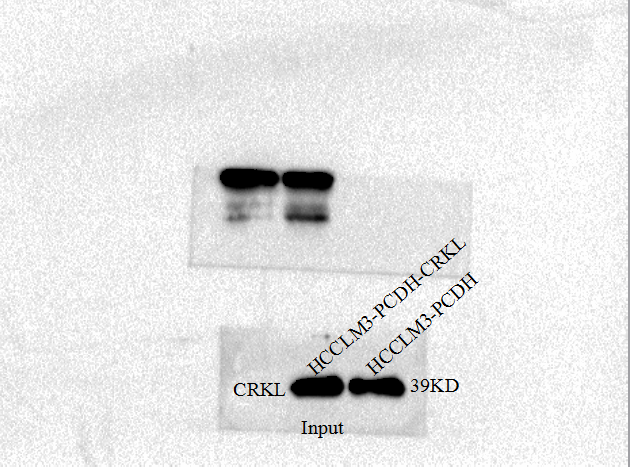


**Panel 51. Blotting images of CRKL for Input**


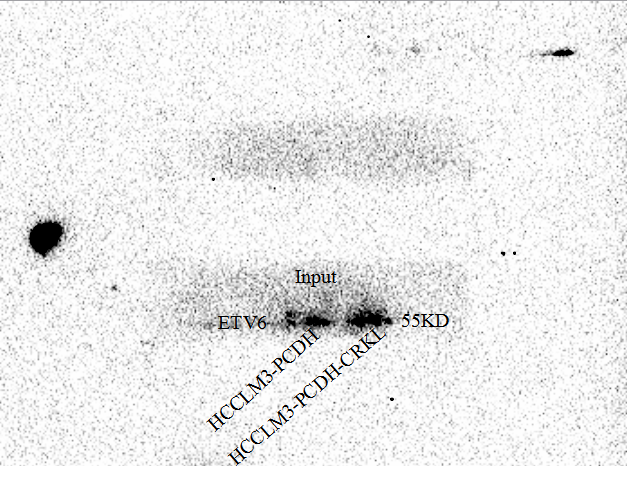


**Panel 52. Blotting images of ETV6 for Input**


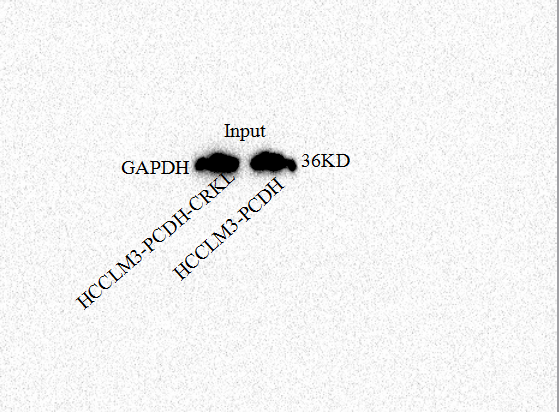


**Panel 53. Blotting images of GAPDH for Input**

**6. Original Western blot images for Fig. 8a: The uncropped western blotting images of ETV6 knockdown in HepG2, HCCLM3 and HuH7 cells were shown in Panel 41, 43, 44, 46, respectively.**

**7. Original Western blot images for Fig. 9a and Fig. 9b: The uncropped western blotting images of CRKL overexpression in HepG2, HCCLM3 and HuH7 cells were shown in Panel 29, 30, 33, 34 for Fig. 9a, respectively. The uncropped western blotting images of CRKL knockdown in HepG2, HCCLM3 and HuH7 cells were shown in Panel 35, 37, 38, 40 for Fig. 9b, respectively.**
